# Analysis of the time course of COVID-19 cases and deaths from countries with extensive testing allows accurate early estimates of the age specific symptomatic CFR values

**DOI:** 10.1371/journal.pone.0253843

**Published:** 2021-08-18

**Authors:** Jessica E. Rothman, David Eidelberg, Samantha L. Rothman, Theodore R. Holford, Douglas L. Rothman

**Affiliations:** 1 Department of Epidemiology of Microbial Diseases, Yale University School of Public Health, New Haven, CT, United States of America; 2 Center for Neurosciences, Institute of Molecular Medicine, Northwell Health, Manhasset, New York, United States of America; 3 Departments of Mathematics and Computer Science, Tulane University, New Orleans, LA, United States of America; 4 Departments of Biostatistics, and Statistics and Data Science, Yale University School of Public Health and Yale University Graduate School of Arts and Sciences, New Haven, CT, United States of America; 5 Departments of Radiology and Biomedical Engineering, Yale University School of Medicine, New Haven, CT, United States of America; University of South Carolina, UNITED STATES

## Abstract

**Background:**

Knowing the true infected and symptomatic case fatality ratios (IFR and CFR) for COVID-19 is of high importance for epidemiological model projections. Early in the pandemic many locations had limited testing and reporting, so that standard methods for determining IFR and CFR required large adjustments for missed cases. We present an alternate approach, based on results from the countries at the time that had a high test to positive case ratio to estimate symptomatic CFR.

**Methods:**

We calculated age specific (0–69, 70–79, 80+ years old) time corrected crude symptomatic CFR values from 7 countries using two independent time to fatality correction methods. Data was obtained through May 7, 2020. We applied linear regression to determine whether the mean of these coefficients had converged to the true symptomatic CFR values. We then tested these coefficients against values derived in later studies as well as a large random serological study in NYC at that time.

**Results:**

The age dependent symptomatic CFR values accurately predicted the percentage of the population infected as reported by two random testing studies in NYC. They also were in good agreement with later studies that estimated age specific IFR and CFR values from serological studies and more extensive data sets available later in the pandemic.

**Conclusions:**

We found that for regions with extensive testing it is possible to get early accurate symptomatic CFR coefficients. These values, in combination with an estimate of the age dependence of infection, allows symptomatic CFR values and percentage of the population that is infected to be determined in similar regions with limited testing.

## Introduction

Knowing the fraction of individuals infected with COVID-19 who will die or require hospitalization is critical for epidemiological modeling and public health policy for mitigating the disease. Unfortunately, it has been difficult to determine the ratio of symptomatic cases that are fatal (case fatality ratio, CFR_actual_) and the fatality ratio for all infections (IFR). The CFR is the number of deaths divided by the number of symptomatic cases in a given time period, and the IFR is the number of deaths divided by the number of infected cases (i.e. cases that may or may not be symptomatic) in a given time period. The major problems in determining these ratios are accurate determination of the number of cases (symptomatic and total) and number of deaths, as well as their age dependence. Early determination during a surge in cases is made more difficult due to the need to correct for the time delay between infection and death. This delay can be up to several months leading to the reported CFR being initially several times lower than the actual CFR even if testing ascertains all symptomatic cases.

The difficulty in obtaining accurate case ascertainment early in a pandemic is demonstrated by the wide range in CFR_actual_ and IFR_actual_ estimates reported through early May 2020, despite sophisticated epidemiological tools being used to correct for missed cases. Based on our meta-analysis presented in the Results section, there was an over 10 fold range in CFR_actual_ and IFR_actual_ estimates reported from top epidemiological groups for the United States and United Kingdom [1–24]. A similar range was reported in an independent meta-analysis [1]. The combination of limited testing and the time dependence of CFR_crude_ represent a major challenge for even the most sophisticated methods that try to correct for missed cases [25].

In this paper we present an alternate method, based on using data from regions with extensive testing, for determining CFR_actual_ values in other regions with limited testing early in a COVID-19 outbreak. We *hypothesized* that even early in their outbreaks, countries that performed extensive testing and case tracking, had ascertained most of their symptomatic cases. We first validated, using a standard time to death correction method and a new method we introduce that does not require this correction, that accurate early calculations of the time corrected CFR_crude_ (CFR_crudetimecorrected_) can be obtained. We then showed by linear regression that the variation of the CFR_crudetimecorrected_ values of the 7 countries we analyzed based on their very low positive to total COVID 19 test ratio could almost completely be explained by three age specific CFR_actual_ values (0 to 69, 70 to 79, and 80 plus years). The values of these age specific CFR_actual_ coefficients were then validated by comparison against serology studies in calculating the percent of the infected population in New York City in late April and early May, as well as comparison with IFR_actual_ values calculated in several regions months after their initial COVID-19 surges.

Our findings have relevance to future outbreaks of COVID, particularly from new variants, by showing that accurate age specific CFR_actual_ values can be obtained early in an outbreak even if extensive testing can only be applied in localized regions due to resource limitations. These values can then be applied to ascertain the actual number of infections and potential mortality in regions with limited testing.

## Methods

### Sources of data

Data for our final analyses were obtained from the Australian, Austrian, German, Iceland, Israeli, South Korean, and New Zealand government websites [8–12, 14, 15, 26, 27]. Data was also obtained from the New York City Department of Health website [17, 28]. We also used the data gathering sites Statista and Worldometer [26, 27] in our preliminary analyses. All analyses were done in R v4.0.1, and all plots were created using the “ggplot2” package.

### Overview of procedures

We present below an overview of the procedures performed in our analysis. Details of the procedures are then presented below.

#### Procedure 1

Using a time from infection to fatality distribution function, based on studies performed in January 2020 in China, we calculated a time corrected CFR crude value (CFR_crudetimecorrected_) from the CFR_crude_ time course of each country using standard methods [20, 22, 24, 29, 30]. The best CFR_crudetimecorrected_ value was determined by goodness of fit to the curve.

#### Procedure 2

We then showed that similar values were obtained using a novel procedure we introduced that does not require knowing the time from infection to fatality distribution function. This method uses only the closed case CFR_crude_ time course.

#### Procedure 3

The ability to accurately calculate CFR_crudetimecorrected_ from very early time course data, was validated by showing that CFR_crudetimecorrected_ values calculated from the full-time courses provided an excellent fit to even the very early portion of the curves.

#### Procedure 4

Using both methods to correct for the time dependence of CFR_crude_ we calculated the overall and age group specific CFR_crudetimecorrected_ for each of the 7 countries for the age groups 0–69 years old, 70–79 years old, and 80 years old and above.

#### Procedure 5

Using linear regression analysis, we found that the large majority of the 8.7-fold CFR_crudetimecorrected_ variation between these countries could be explained by three constant CFR_actual_ coefficients for the 0 to 69, 70–79 and 80–89 groups.

#### Procedure 6

We validated these coefficients by predicting the COVID-19 infected population in New York City in late April and Early May, which we found had excellent agreement with serology studies. In addition, the coefficients are shown to be in excellent agreement with values ascertained several months later after the initial COVID 19 surges had subsided in several regions.

**Table:** 

Definitions	
t	A given day after the start of the outbreak
j	Day person got infected; represents the start of a new cohort
C	Case: only individuals who are symptomatic
I	Infection: individuals who are symptomatic or asymptomatic
n_C_(j)	Number of new cases on day j
N_C_(t)	Cumulative number of cases on day t after the start of the outbreak:
	$N_{C}(t)=\sum_{j=1}^{t}n_{C}(j)$
n_D_(j)	Number new fatalities (deaths) on day j
N_D_(t)	Cumulative number of fatalities (deaths) on day t after the start of the outbreak for the total number of cohorts, J:
	$N_{D}\;(t)=\sum_{j=1}^{J}n_{D_{j}}(t)$
N_I_(t)	Cumulative number of infections on day t after the outbreak
N_CC_(t)	Cumulative number of closed cases (died or recovered) on day t
N_R_(t)	Cumulative number of recovered cases on day t
CFR_crude_	The uncorrected, often referred to as naïve/ crude, measured ratio of cumulative number of fatalities divided by the cumulative number of cases on a given day:
	$CFR_{crude}(t)=\frac{N_{D}(t)}{\sum_{j=1}^{J}{n_{C}}_{j}}$
CFR_closedcase_	Same as CFR_crude_ but measured using only data from closed cases (either recovered or dead) given by [N_D_(t)/N_CC_(t)]
CFR_crudetimecorrected_	The corrected case fatality rate (CFR_crudetimecorrected_) is the reported CFR_crude_ corrected for the time delay between diagnosis and fatality
CFR_actual_	The true CFR value when all symptomatic cases are detected
CFR_crudetimecorrected_(0–69)	Case fatality ratio for the age group 0 to 69 years old
CFR_crudetimecorrected_(70+)	Case fatality ratio for the age group 70+
CFR_crudetimecorrected_(70–79)	Case fatality ratio for the age group 70–79
CFR_crudetimecorrected_(80+)	Case fatality ratio for the age group 80+
CFR*_crudetimecorrected()_	Contribution of an age group to the total CFR_crudetimecorrected_
	CFR_crudetimecorrected_ = CFR*_crudetimecorrected(0–69)_ + CFR*_crudetimecorrected(70+)_
p(0–69)	Percentage of infected population between age 0 and 69.
p(70–79)	Percentage of infected population between age 70 and 79.
p(80+)	Percentage of infected population 80 years and older
p(70+)	Percentage of infected population 70 years and older
IFR	The infection fatality ratio (IFR) given by the ratio of cumulative number of fatalities divided by the cumulative number of infected [N_D_(t)/N_I_(t)]; can only be achieved if the entire population is tested accurately.
*f*_D_(t)	Probability density function of fatality at t days after diagnosis
*F*_D_(t)	Cumulative distribution function obtained from *f*_D_(t)

### Calculations

#### Time correction of CFR_crude_(t) for the delay between diagnosis and fatality

We used two independent methods to estimate the corrected CFR. In one method we corrected the reported CFR_crude_(t) for the time delay between diagnosis and fatality based on previously reported approaches [6, 7, 22, 24, 30–33]. In the second, we used closed case CFR_crude_(t) time courses, which does not require knowing the time to fatality distribution function.

In the first method, we implemented a time delay to fatality correction method using a time delay to death distribution function f_D_ derived from reported log-normal fits of data obtained from China, between December and late January, of the percentage of fatalities of COVID-19 patients per day after diagnosis [22, 24, 29, 30, 33]. Data was used only from patients who were hospitalized outside of Hubei province to avoid the potential problem that adequate medical care was likely not available within the province, and especially in Wuhan, early in the outbreak [6, 32, 33]. For the cohort of cases diagnosed on day j_,_ the *f*_*D*_ at day t is described by,

$${f_{D}(t-j)}_{}=Lognormal(\log\;Mu,\log\;SD)$$ 1

The calculated cumulative number of fatalities from the cohort diagnosed on day j on day t was calculated from the cumulative distribution (*F*_D_) which is the integral of Eq [1] from day j to day t multiplied by the number of new cases on day j and the corrected CFR,

$$n_{D_{j}}(t)={CFR}_{crudetimecorrected}*n_{C_{j}}*F_{D}(t-j)$$ 2

where *t*>*j*.

We note that Eq [2] is equivalent to a convolution integral of *f*_*D*_(t-j) with a delta function centered at day j with an area of CFR*n_Cj_.

The value of the CFR_crudetimecorrected_ was then calculated by adjusting the value of the CFR_crudetimecorrected_ in Eq [2] until the calculated CFR_crude_(t) on the last day of the outbreak analyzed was equal to the reported value.

#### Calculation of CFR_crudetimecorrected_ from closed case CFR_crude_ time courses

The second method was based on our observation that in all countries analyzed the closed case CFR (see definitions) converged to a near constant value well prior to the value of CFR_crude_. A closed case is defined as a case that has been designated as recovered or has died. The advantage of this method is that it does not require knowledge of the time to death distribution function, only that convergence has been achieved based on time course analysis. As shown in S3 Fig, provided that the median times to fatality and for recovery stay approximately constant during the outbreak, the closed case CFR_crude_(t) will converge to the final value prior to the CFR_crude_(t).

#### Assessment of the sensitivity of the correction factor to the assumed input function, *f*_D_

The function f_D_ used for the first time correction method, was based on reports of the measured onset (day of positive test) to fatality distributions for Chinese patients outside of Wuhan who were infected in December and January by Linton et al. and Mizumoto et al. [30, 33]. These investigators modeled the distributions as Log-normal functions that were corrected for right censoring (fatalities missed due to the limited patient observation time). The best fitting distributions from these sources were very similar, with Linton reporting a best fit median of 13.2 days with a 95% CI of 11.5 to 15.3 days, and Mizumoto et al. reporting a best fit median (estimated from their reported log-mean value) of approximately 13 days [30, 34, 35].

Because these results were all obtained early in the pandemic and before the final outcome of all the patients studied was known, we tested the sensitivity of our time to death correction to the range of variation in the median and shape of the published distributions. For the median (50% of fatalities have occurred) we used values of 14, 17, and 21 days to cover the full range of reports. The studies which used gamma fits reported a very similar shape of the distribution to the studies that fit the data to a lognormal distribution, equivalent to a logSD of approximately 0.50 as reported by Mizumoto [34, 35]. Goodness of fit was determined by calculating the least squares total residual by squaring the differences between our calculated CFR_crude_(t) (using the CFR_crudetimecorrected_) and the reported CFR_crude_(t) values, and then summing those squares. The simulations were performed using data from Germany due to the much larger number of infected subjects, which minimizes small number statistical simulations. We found that there were relatively small variations in goodness of fit and CFR_crudetimecorrected_ values calculated over the range of 14, 17, and 21 days and for each value of the median varying logSD from 0.25 to 0.75, with the best fit being for a median of 14 days and a logSD of 0.50. We then used these values in analyzing data from the other countries.

#### Calculation of median and range of age dependent CFR_crudetimecorrected_ values

We calculated the values of CFR_crudetimecorrected_ for the age range of 0–69, 70–79, and 80 and above (CFR_crudetimecorrected_(0–69), CFR_crudetimecorrected_(70–79), CFR_crudetimecorrected_(80+). As described below, we then validated these values using linear regression in which we plotted the age specific components of the CFR_crudetimecorrected_ for each country (e.g. CFR*_crudetimecorrected(70+)_) versus the population percentage in the age range and showed that they could be fit by constant coefficients.

#### Determination by linear regression of whether the range of measured age specific CFR_crudetimecorrected_ values for each country could be fit by three constant age specific CFR_actual_ values (0 to 69, 70 to 79, 80+)

Despite the countries examined all having a high ratio of total tests to positive cases, there was a large variation in their CFR_crudetimecorrected_ values, from 0.58 to 5.0 (Table 2). To test whether this variation could be explained by constant age dependent CFR_actual_ coefficients, we first performed a simple linear regression of the proportion of CFR_crudetimecorrected_ due to the 70+ group range (CFR_crudetimecorrected_*_(70+)_) versus the proportion of the infected population in this age for each country (p(70+)).

If CFR*_crudetimecorrected_ is determined by the age specific CFR_actual_ coefficients, as opposed to variations in testing or other factors not related to the disease, the value of CFR*_crudetimecorrected(70+)_ is related to CFR_actual_(70+) by the following relationship:

$${CFR}_{crudetimecorrected(70+)}^{*}={CFR}_{actual}(70+)*p(70+)$$ 3

To determine how much of the variation in CFR_crudetimecorrected(70+)_ between countries can be explained by a single value of CFR_actual_*(70+), we calculated the R^2^ of the least squares regression. We also compared the value of the slope to the value of CFR_crudetimecorrected_(70+) determined from the mean values of the countries analyzed.

We further broke down CFR_crudetimecorrected(70+)_ to understand how much of the remaining variation could be explained by using separate constant CFR_actual_ coefficients for the population in the 70–79 age group and 80+ age groups respectively using Eq [4]:

$$\begin{array}{l} {CFR}_{crudetimecorrected(70+)}^{*}\\ \;={CFR}_{actual}(70-79)*p(70-79)+{CFR}_{actual}(80+)*p(80+) \end{array}$$ 4

To allow the goodness of fit to be shown in one graph we normalized CFR_crudetimecorrected(70+)_ to the mean value of $\frac{p(80+)}{p(70+)}$ between countries of 0.40 (Table 3). The normalization used each country’s measured value of CFR_crudetimecorrecte_d (80+) and CFR_crudetimecorrected_(70–79).


$$\begin{array}{l} {CFR}_{crudetimecorrected(70+)A}^{*}\\ \;={CFR}_{crudetimecorrected}(70-79)*\left(1-(\frac{p(80+)}{p(70+)})\right)\\ \;+{CFR}_{crudetimecorrected}(80+)*\left(\frac{p(80+)}{p(70+)}\right) \end{array}$$ 5

$$\begin{array}{l} {CFR}_{crudetimecorrected(70+)A}\\ \;={CFR}_{crudetimecorrected}(70-79)*0.60\\ \;+{CFR}_{crudetimecorrected}(80+)*0.40 \end{array}$$ 6

#### Calculation of CFR_actual_ for New York and regions of China based on the age distribution of positive cases in the population and the age specific CFR_actual_ values determined from the age specific CFR_crudetimecorrected_ coefficients

We calculated the CFR_actual_ for New York City and regions of China (as reported by the WHO) using the following equation:

$${CFR}_{actual}={CFR}_{crudetimecorrected}(0-69)*p(0-69)+{CFR}_{crudetimecorrected}(70-79)*p(70-79)+{CFR}_{crudetimecorrected}(80+)*p(80+)$$ 7

where **p()** is the proportion of the population in the relevant age groups in China or New York City. The age specific coefficients were determined from the 7 countries analyzed as described above.

#### Calculation of the percentage of the adult population of New York City that has been infected with COVID-19 on April 22, 2020

We used Eq [3] to calculate CFR_actual_ for New York City using the reported percentages of cases above 0–69, 70–79, and 80+ years. Values were interpolated from the age groups reported on the New York City public health site [17, 36].

To estimate the total number of infected individuals in the population, we divided the time corrected number of fatalities by the IFR [17]. The IFR was calculated from the CFR_crudetimecorrected_ values based on the assumption that the CFR_actual_ was achieved in the countries analyzed. A factor of 2 was then used to convert the CFR to IFR based on reports of half of all COVID-19 cases being asymptomatic and may have escaped detection [17, 37–39].

A time correction factor (CF_t_) of 1.74 was calculated from the new cases per day as described above. We assumed based upon a relatively constant number of tests per day over this period that the captured cases would be proportional to the total number of new cases per day in the population [17, 38, 39].


$$Number\;of\;infections=\frac{N_{D}(t)*{CF}_{t}}{\frac{1}{2}C{FR}_{actual}}$$ 8

For the total number of fatalities, we used the confirmed cases to attain a minimum estimate; we then added probable fatalities for a maximum estimate. To determine the percent of the adult population infected, we then divided the maximum and minimum number of infections by the number of adults (over age 18) in New York City [38]. The adult population number was used due to the random testing not including children, who are known to have a much lower symptomatic and total infection rate than adults [8–11, 14]. We also compared our calculations with other models using their reported IFR values (Table 1) and Eq [8].

**Table 1:** Reported CFR_crude_, CFR_actual_, and corrected IFR values for China, the United Kingdom and the United States. The table summarizes CFR_crude_ for each country region at the time of the report, calculated CFR_actual_ and IFR values through early May 2020. Details are available in the cited references [2, 4, 6–14, 16, 19, 21–24, 32, 33, 40, 41]. For the USA and UK the CFR_crude_ on April 15, 2020 is listed. Studies are listed by their first author or by the location of the modeling group that reported them.

Report	CFR_crude_	(CFR_actual_)	IFR	Region
Bendavid et al. [2]	3.90%	0.18%*	0.12–0.2%	Santa Clara County, California
Oxford [21]	16.7%	0.25%*	0.1–0.36%	United Kingdom
DHHS model early April 2020	5.0%	0.25%		United States
DHHS model mid- April 2020	5.0%	0.50%		United States
Ioannidis et al. [13]	5.0%	0.26%*	0.13%	United States
CDC May 2020 [40]	5.0%	0.2%		United States
JHU [23]	5.0%	0.60%		United States
Pei and Shaman [18]	5.0%	1.1%*	0.56%	United States
Modi et al. [19]	10.20%	1.0%*	0.50%	New York City
Imperial College [3]	16.7%	1.8%*	0.90%	United Kingdom
Mizumoto et al. [33]	1.80%	0.90%		China (Hubei province)
Mizumoto et al. [33]	0.43%	0.90%		China (outside Hubei)
Li et al. [41]	3.60%	0.90%	0.40%	China
Russell et al. [24]	3.50%	1.10%	0.50%	China
Verity et al. [22]	3.70%	1.38%	0.60%	China
Wu et al. [32]	4.5%	1.40%		China (Wuhan)
Wu et al. [4]	0.85%	0.85%		China (outside Wuhan)
Hauser et al. [6]	2.40%	3.00%		China (Hubei province)
Baud et al. [7]	3.60%	5.60%		China
Present Work	1.41%	1.58%		Australia
	3.89%	4.25%		Austria
	4.36%	5.00%		Germany
	0.56%	0.58%		Iceland
	1.47%	2.16%		Israel
	2.28%	2.65%		South Korea
	1.41%	1.55%		New Zealand
	3.50%	2.19%**	1.10%**	China (Feb 11, 2020)
	10.20%	3.60%**	1.80%**	Adults New York City (April 22, 2020)

**: Not time corrected based on case data.

*: estimated from IFR, **: calculated from age dependent CFR coefficients from present manuscript. Abbreviations: CDC: Center for Disease Control USA; DHHS: Department of Health and Human Services, USA; Oxford: Oxford College, U.K.; Imperial College: Imperial College, U.K.

#### Simulation of the closed case CFR(t)

To understand the basis for the apparent early convergence of the closed case CFR_crude_ to the CFR_crudetimecorrected_ value, we calculated the cumulative number of recoveries versus day after the outbreak using the above approach for calculating cumulative fatalities (S4 Fig). Case per day data from South Korea and Germany were used in the simulations. Based on recent reports from Verity and Bi and earlier work by Ghani with SARS, the distribution function for time to recovery *f*_R_ is similar to that for fatality but with a median shifted several days later and a less right skewed distribution [22, 29, 31]. Based on these reports, we used a lognormal *f*_*R*_ with a logSD of 0.25 and examined the effect of the median shift on the convergence to the CFR_crudetimecorrected_ value of closed case CFR(t) curves [22, 31].The closed case CFR(t) was calculated using the following formula,

$$closed\;case\;CFR(t)=\frac{N_{D}(t)}{N_{R}(t)+N_{D}(t)}$$ 9

## Results

### Meta-analysis of reported IFR and CFR values for COVID 19 as of early May 2020

Table 1 presents values reported for the UK and USA from epidemiological laboratories of CFR_actual_ and IFR_actual_ for COVID-19 as of early May 2020. Values reported for China are also included. For the US and UK, there was a 10-fold range in reported values, and a 6-fold range for China. The Table also presents the uncorrected CFR (CFR_crude_) for each country/region. For China, the UK, and USA they were up to several fold higher than the calculated values of CFR_actual_ demonstrating inadequate ascertainment of total cases (Table 1) [11, 21, 22, 26].

#### Increase in the reported CFR_crude_(t) versus time after the start of the outbreak in 7 countries

**Fig 1:**
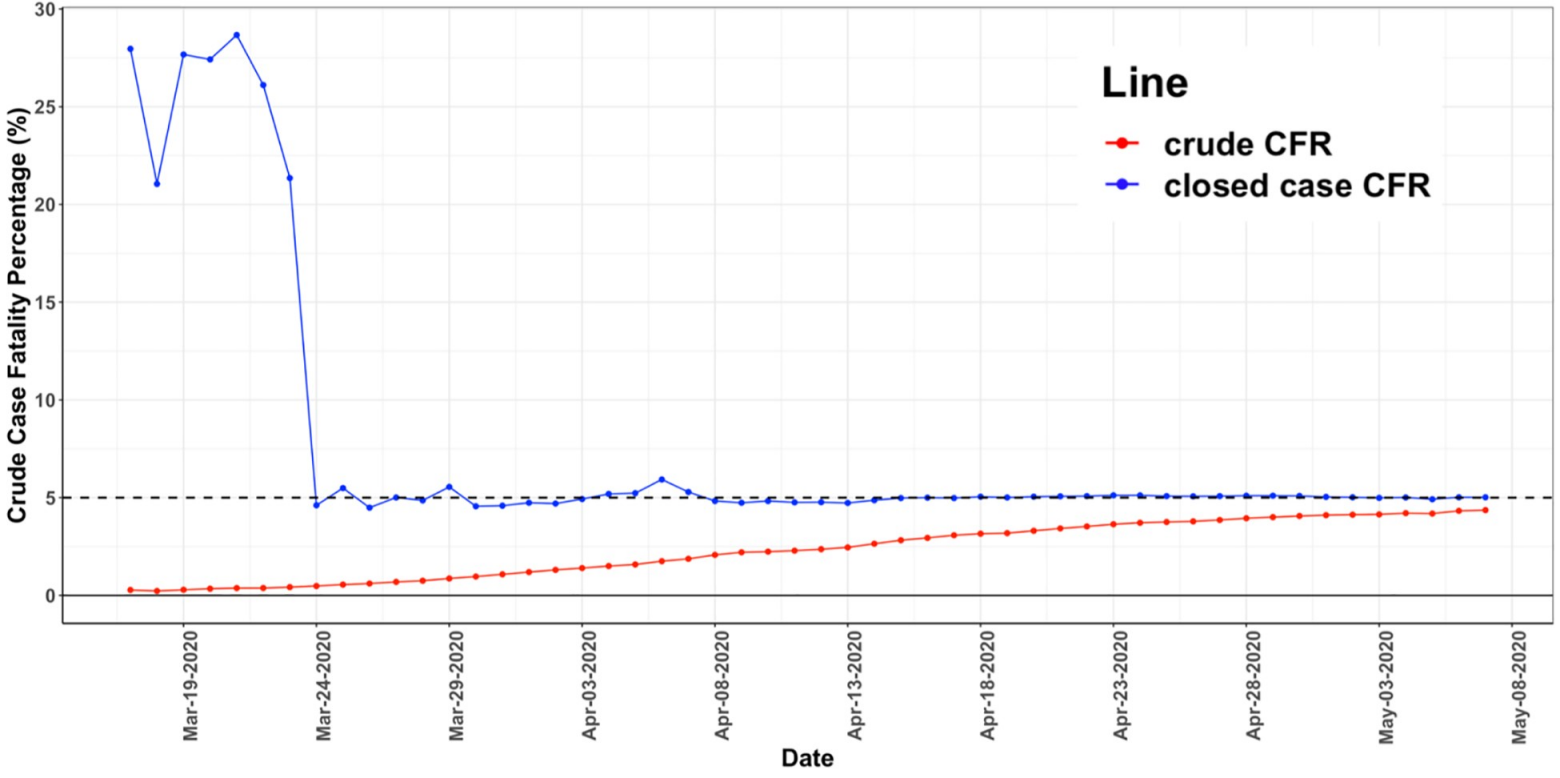
CFR_crude_(t) and CFR_closedcase_(t) versus time for Germany. The bottom curve (red) shows CFR_crude_(t) plotted versus day after outbreak. The top curve (blue) shows the same for CFR_closedcase_(t). CFR_crude_(t) increases over this period from a value of 0.12% to a value of 4.36%. It is seen that CFR_closedcase_(t) converges to the projected true of CFR_crude_ earlier than the CFR_crude_(t) curve itself.

We found in all countries examined that the reported CFR_crude_ increased throughout the COVID-19 outbreak. As shown in Fig 1 the value of the reported CFR_crude_(t) for Germany rose from a low value of 0.12% on March 10, 2020 to a value of 4.36% on May 7, 2020. Our estimate of the final CFR of 5.0% is shown as a dashed horizontal line. The values shown are plotted from 10 days after the first 100 cases were reported to avoid large fluctuations due to the small numbers of initial fatalities. In S2 Fig, we show that the CFR_crude(t)_ versus day curves for Austria, Australia, Iceland, Israel, and New Zealand exhibited the same behavior of a large early underestimate of the final value.

#### The reported closed case CFR_crude_ time course converges before the CFR_crude_ time course to its final value

We found that for the countries we examined, the closed case CFR_crude_ value converged to a constant value prior to the CFR_crude_ time course. In Fig 1, we plot CFR_closedcase_(t) and CFR_crude_(t) curves from Germany. The curves show CFR_crudeclosedcase_ had converged 48 days prior to May 7, 2020, while the CFR_crude_ continued to increase. S1 Fig shows that a similar convergence to a stable value also occurred for Australia, Austria, Iceland, Israel, New Zealand, and South Korea prior to convergence to its actual value at the end of the outbreak.

#### Estimation of the final value of CFR_crude_, using the standard time correction method and from the closed case CFR after convergence

As shown in Table 2, the closed case CFR convergence and standard time correction methods gave similar results for all of the countries examined. This finding supports that CFR_closedcase_ converged early to close to the actual CFR_crude_ value.

**Table 2:** Comparison of the time corrected CFR_crude_ values calculated using the closed case convergence method versus the standard time to fatality time correction method [8–12, 14, 15, 26].

Country	Closed Case CFR	CFR_crudetimecorrected_
Australia	1.58	1.42
Austria	4.26	4.20
Germany	5.02	5.05
Iceland	0.57	0.58
Israel	2.16	1.72
New Zealand	1.55	1.51
South Korea	2.65	2.32

#### Assessment of the accuracy of early determination of CFR_crudetimecorrected_

**Fig 2:**
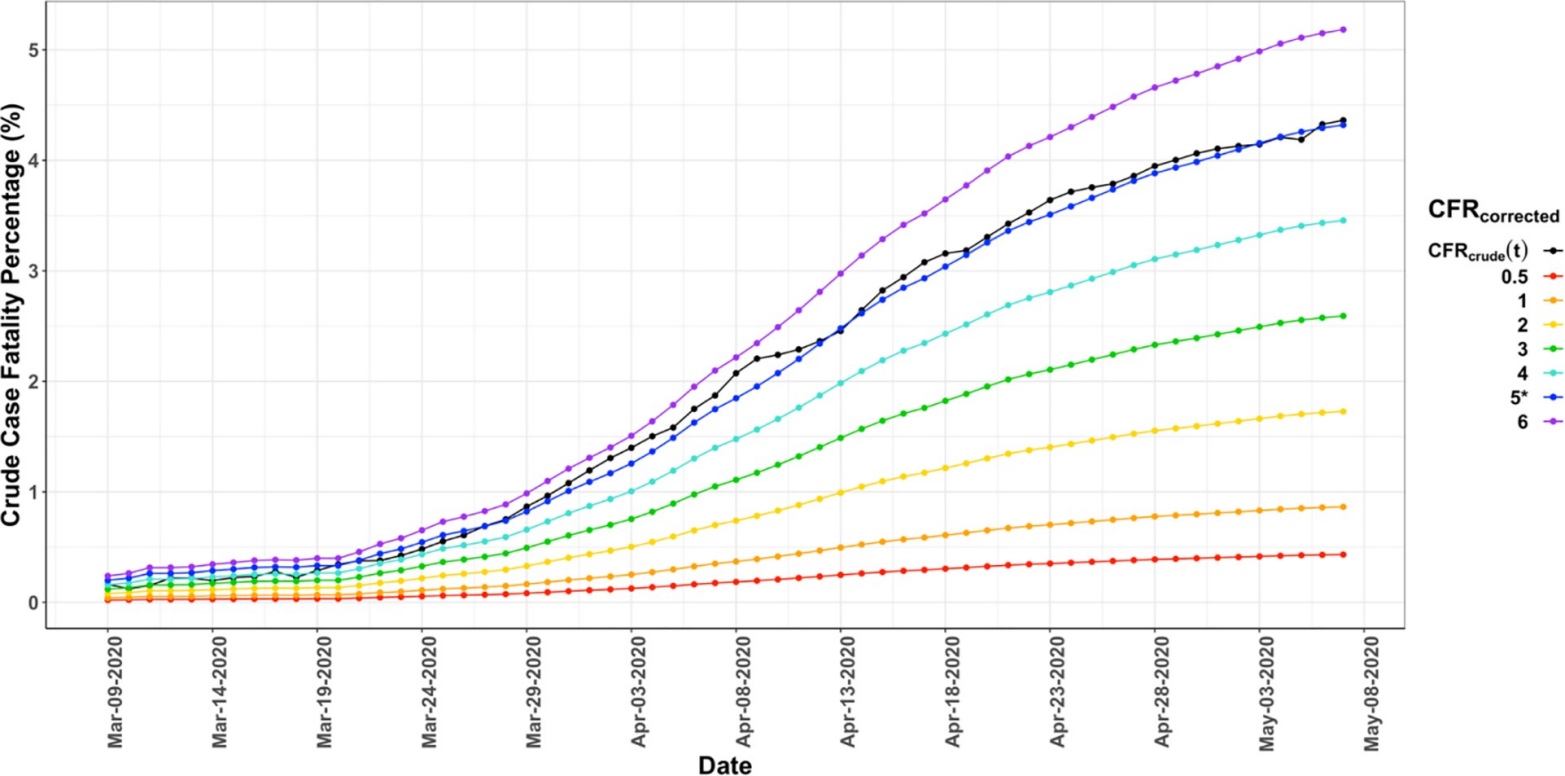
Simulated and reported CFR_crude_(t) versus time curves for Germany. The reported CFR_crude_(t) curve is plotted in black. Even though the reported CFR_crude_(t) curve rises by more than 10-fold, it is well matched throughout the duration by the simulated CFR_crude_(t) curve (blue) using the CFR_crudetimecorrected_ value of 5.0% determined from the entire time course. Therefore, even early in the outbreak, when CFR_crude_(t) was 10-fold lower than on May 7, 2020 (the last day used) the time correction method would have accurately predicted the true CFR_crude_(t) value.

To determine the accuracy of applying the time correction and closed case convergence methods early in an outbreak we simulated the CFR_crude_(t) time courses using the CFR_crudetimecorrecte_(t) values (Table 2) calculated from the entire curves. As shown in Fig 2, using the example of Germany, the curve generated using the CFRcrude(t) versus time curves calculated using the CFR_crudetimecorrected_ value of 5.0 (blue) matches the actual data (black) well throughout the entire time course. Similar results were found for the other countries (see SI for fits). These results demonstrate that even very early in an outbreak an accurate value of CFR_crude_ can be determined.

#### Determination of age specific CFR_actual_ coefficients

We calculated for each country the CFR_crudetimecorrected_ coefficients in the age ranges 0 to 69, 70–79, and 80–89 (see Methods). We then tested whether the large variation in values of CFR_crudetimecorrected_ between these countries could be explained by the distribution of the infected population in these age groups We chose these age ranges because of early reports that the majority of fatalities were in older age groups [24, 42]. As shown in Table 3, the age group specific values of CFR_crudetimecorrected_ increased rapidly with age and were between the countries studied.

**Table 3:** Age specific fractions of cases, age specific corrected CFR, and contributions of each age group to the overall corrected CFR for each country.

**Country**	**p(0–69)**	**p(70–79)**	**p(70+)**	**p(80+)**	**p(80+)/ p(70+)**	**CFRc(80+)**	**CFRc(70–79)**	**CFRc(70+)**	**CFRc(0–69)**
**Australia**	86.11%	10.54%	13.89%	3.35%	24.09%	27.50%	3.95%	9.62%	0.28%
**Austria**	81.51%	8.62%	18.49%	9.87%	53.37%	24.23%	12.62%	18.80%	0.95%
**Germany**	81.12%	8.87%	18.88%	10.01%	53.00%	31.83%	13.06%	23.01%	0.81%
**Iceland**	95.38%	3.40%	4.62%	1.22%	26.51%	18.95%	5.13%	8.79%	0.18%
**Israel**	91.00%	5.30%	9.00%	3.70%	41.11%	35.30%	9.49%	20.10%	0.39%
**New Zealand**	92.21%	7.79%	7.79%					17.08%	0.22%
**South Korea**	88.90%	6.59%	11.10%	4.51%	40.59%	28.07%	12.05%	18.56%	0.63%
**Mean**	88.03%	7.30%	11.97%	5.44%	39.78%	27.65%	9.38%	16.57%	0.49%
**SD**	5.40%	2.41%	5.40%	3.65%	12.52%	5.72%	3.97%	5.35%	0.30%
**95% CI of Mean**						(21.64%, 33.65%)	(5.22%, 13.55%)	(10.95%, 22.18%)	(0.18%, 0.81%)
**Country**	CFRc*_(*0–69)_	CFRc*_(70+)_	**CFRc**	CFRc*_(70+)_/ **CFRc**	**CFRc(70+)**_A_	CFRc*_(0–69)A_	CFRc*_(70+)A_	**CFRc**_A_	CFRc*_(70+)A_/ **CFRc**_A_
**Australia**	0.24%	1.34%	1.58%	84.62%	13.37%	0.24%	1.86%	2.10%	88.43%
**Austria**	0.60%	3.54%	4.25%	83.29%	17.26%	0.60%	3.05%	3.76%	81.13%
**Germany**	0.66%	4.34%	5.00%	86.84%	20.57%	0.66%	3.88%	4.54%	85.50%
**Iceland**	0.21%	0.37%	0.58%	63.82%	10.66%	0.21%	0.49%	0.70%	70.11%
**Israel**	0.38%	1.81%	2.16%	83.75%	19.81%	0.38%	1.78%	2.16%	82.55%
**New Zealand**	0.22%	1.33%	1.55%	85.71%	17.08%	0.22%	1.33%	1.55%	85.71%
**South Korea**	0.59%	2.06%	2.65%	77.73%	18.46%	0.59%	2.07%	2.66%	77.80%
**Mean**	0.41%	2.11%	2.54%	80.82%	16.74%	0.41%	2.07%	2.50%	81.60%
**SD**	0.20%	1.37%	1.57%	8.04%	3.55%	0.20%	1.11%	1.30%	6.14%
**95% CI of Mean**					(14.1%, 19.4%)				

Definitions: **p()** is the proportion of the population in the relevant age group; **CFRc(80+)** is the CFR_crudetimecorrected_ for cases 80 years old and above; **CFRc(70–79)** is the CFR_crudetimecorrected_ for cases 70–79 years old; **CFRc(70+)** is the CFR_crudetimecorrected_ for cases 70 years old and above; **CFRc(0–69)** is the CFR_crudetimecorrected_ for all cases 69 years old and below; **CFRc**_**(70+)**_ is the contribution to the overall CFR_crudetimecorrected_ (CFRc) from all cases 70 years old and above; **CFRc**_**(0–69)**_ is the contribution to CFR_crudetimecorrected_ from all cases 69 years old and below. The **subscript A** refers to CFRc*_(70+)_ values corrected to have a fraction of 40% of cases 80 years old and above. The value was chosen to match the mean from all countries except New Zealand (which has not reported this value and therefore it was assumed to be the same as the mean of the other countries). Thus CFRc*_(70+)A_ is calculated by multiplying CFRc(70+)_A_ by p(70+). Please see Eqs 5–9 for further explanation as to how values were calculated. Data was obtained from the following references [8, 9, 11, 12, 14, 15, 26, 27].

#### Determination of whether case age distribution accounted for the differences in CFR_crudetimecorrected_ between countries

Even though all of the countries studied had extensive testing there was a large variation in their overall values of CFR_crudetimecorrected_ (Table 2). To determine whether this variation was due to differences in their age distribution, or other factors such as the percentage of case ascertainment, we performed a linear regression of age group specific CFR_crudetimecorrected_ for each country versus the percentage of the population in the 70+ age range. In the analysis the CFR_crudetimecorrected_ values calculated for each country and decomposed it into two age specific components,

$${CFR}_{crudetimecorrected}={CFR}_{crudetimecorrected(0-69)}^{*}+{CFR}_{crudetimecorrected(70+)}$$ 10

As described in the Methods, the values of CFR*_crudetimecorrected(0–69)_ and CFR_crudetimecorrected(70+)_ are related to the age specific CFR coefficients by,

$${CFR}_{crudetimecorrected(0-69)}^{*}={CFR}_{crudetimecorrected}(0-69)*p(0-69)$$ 11

And

$${CFR}_{crudetimecorrected(70+)}^{*}={CFR}_{crudetimecorrected}(70+)*p(70+)$$ 12

**Fig 3:**
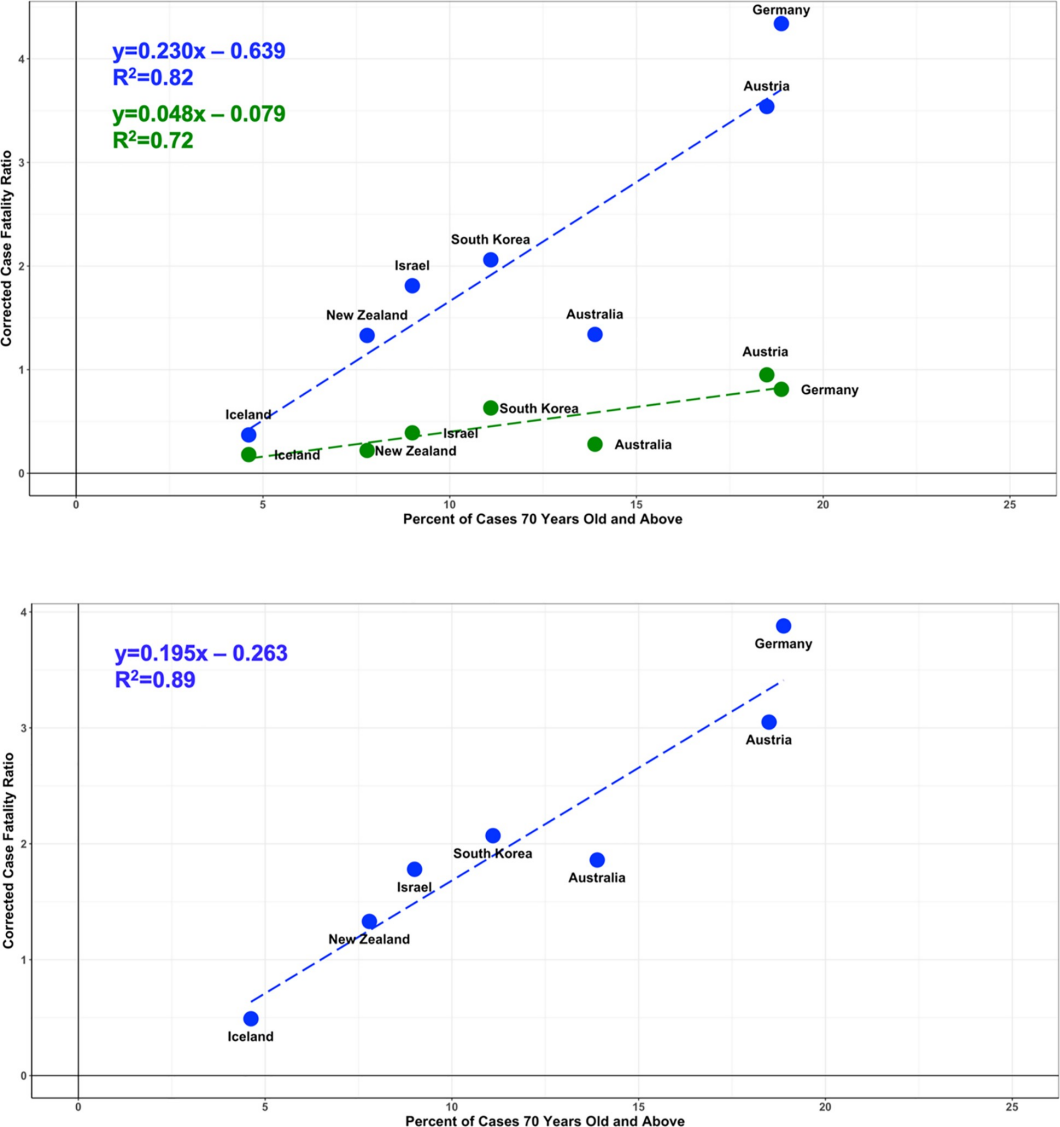
Linear regression analysis of CFR*_crudetimecorrected(70+)_, CFR*_crudetimecorrected(70+)A_, and CFR*_crudetimecorrected(0_−_69)_ versus percent of cases 70 years old and above (p(70+)). **A** shows a plot of CFR*_crudetimecorrected(70+)_ (blue) and CFR_crudetimecorrected(0_−_69)_ (green) for each country versus the percent of cases 70 years old and above (p(70+)). It is seen that for all countries the CFR_crudetimecorrected(70)_ term explains the large majority of CFR_crudetimecorrected_ (81% +/- 8%). The majority of the variance in CFR_crudetimecorrected(70)_ is explained by cases 70 years old and above (R^2^ = 0.82). **B** shows a plot of CFR_crudetimecorrected(70+A)_ (blue) for each country. The value of cCFR_70+A_ for each country was calculated by adjusting the fraction of cases in the 70 and over group who are 80 years old and above to be 40% (p(80+)/p(70+) = 0.40), which is the mean of the countries examined (Table 3). The higher fraction of the variance explained by age for CFR_crudetimecorrected(70+A)_ (R^2^ = 0.89) indicates that the percentage of the population 80 years and older are an important factor in determining the average population value of CFR_crude_.

Fig 3A shows a linear regression of the term CFR*_crudetimecorrected(70+)_ plotted against the fraction of the infected population 70 years and older (blue points). The term CFR*_crudetimecorrected(70+)_ contains all deaths for cases 70 years old and above. The best fit slope corresponds to the mean value of CFR_crudetimecorrected_(70+). As seen in the plot a good linearity of fit is observed with 82% of the variation explained. It is seen that for all countries the CFR*_crudetimecorrected(70+)_ term explains the large majority of CFR_crudetimecorrected_ (81% +/- 8%, Table 3).

To see if the remaining variation could also be explained by age distribution we adjusted the value of CFR_crudetimecorrected_(70+) measured for each country, for the fraction of their case population 80 years and older p(80+) and taking into account the higher CFR_crudecorrected_ in the 80+ group (see Methods). As seen in Fig 3B, taking into account the higher CFR_actual_ of the 80+ group further improved the regression to where 89% of the variation was accounted for.

The contribution to CFR_crudetimecorrected_ from cases 69 years old and younger showed a weak dependence on p(70+) (slope = 0.05, R^2^ = 0.72), which may reflect that countries with a higher percentage of cases in the 70+ group also have a higher percentage in the 60–69 year old group which has also been shown to have an elevated risk of death from COVID-19.

#### Estimation of CFR_actual_ for China as of February 11, 2020 and New York City as of April 22, 2020

We estimated CFR_actual_ for China using the mean age specific CFR_crudetimecorrected_ coefficients, the case population distribution reported for China (p(0–69): 88%, p(70–79): 9%, p(80+): 3%) (39) and Eq [5] in the methods. The CFR_actual_ obtained was 2.2% with a 95% CI of 1.54–2.85%. Due to the greater percentage of the infected population in the 70+ range in NYC (p(70–79): 9%, p(80+): 8%) we calculated a higher CFR_actual_ value for NYC of 3.60% with a 95% CI of the mean: 2.73%-4.47%.

#### Estimation from serological studies of COVID-19 from New York City of the population IFR and comparison with the calculated CFR_actual_ value

We tested the calculated CFR_actual_ for NYC against serological estimates of the percent of the adult population infected. We used the number of deaths reported in NYC as of April 22, 2020 and applied a time correction based on case per day data. We converted the CFR_actual_ values to IFR values using estimates of percent asymptomatic cases from the Diamond Princess in which all passengers were tested (Methods).

**Fig 4:**
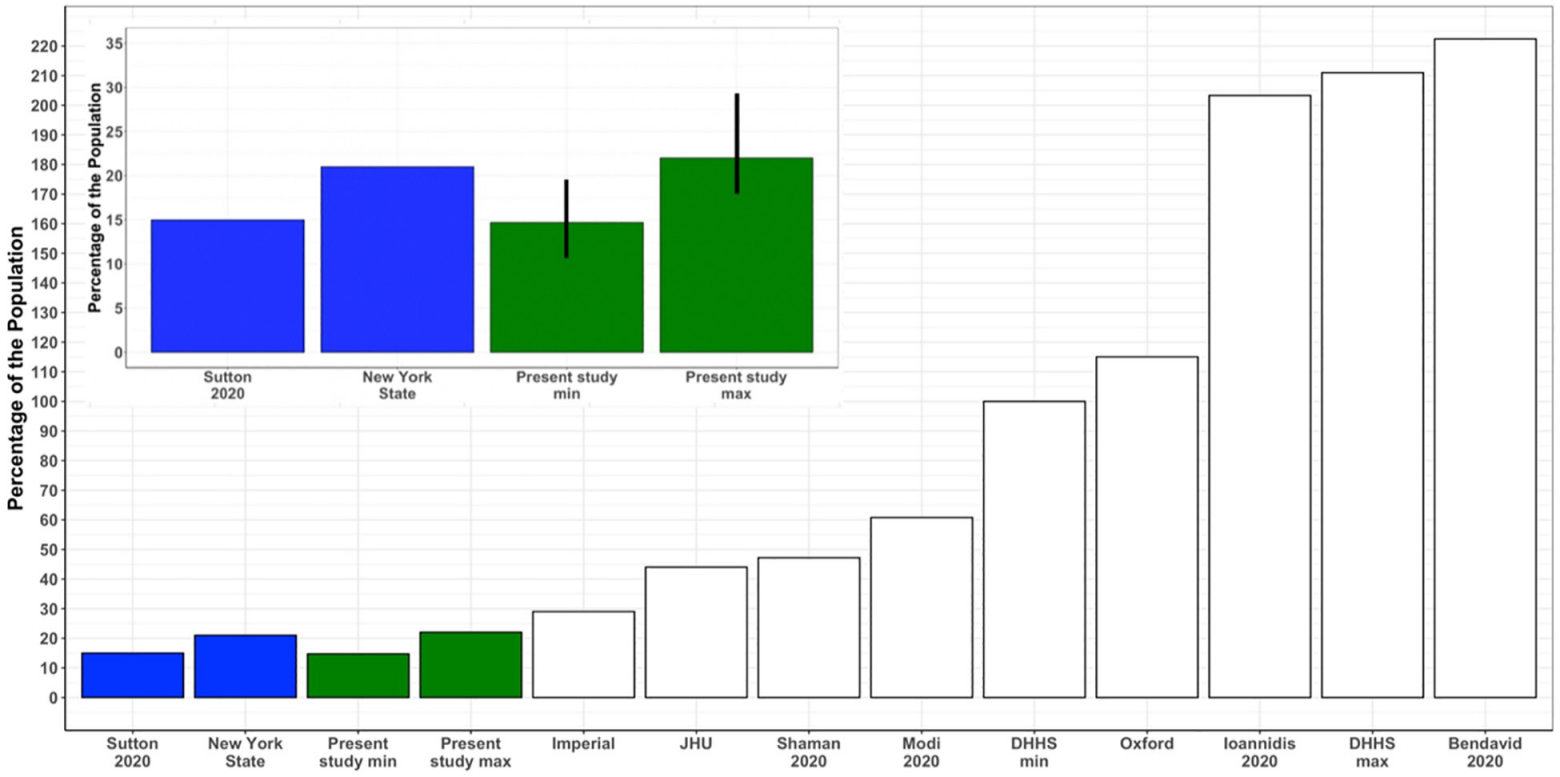
Reported percentage of New York City adults infected with COVID-19 versus percentage calculated from our and other reported IFR values prior to May 7, 2020. As shown in the inset, the predicted maximum and minimum percent of the population in New York City infected with COVID-19 is within the range determined from random adult serological testing [42, 43]. For comparison, we plotted the percentage infected using the IFR values in Table 1.

The inset in Fig 4 shows our minimum and maximum calculated values (green bars) of 14.69% (95% CI of mean: 11.85%-19.43%) and 22.05% (95% CI of mean: 17.75%- 29.10%). These values are seen to be in agreement with serological studies in late April and early May that randomly tested individuals in the NYC adult population of 15.3% and 21%, respectively (blue bars) [42, 43]. In contrast the majority of reported IFR and CFR values reported up to early May 2020, predicted much higher infection percentages, as shown in the main figure.

## Discussion

Rapid determination of the actual symptomatic CFR and IFR values early in a COVID outbreak is hampered by the lag between case detection and fatality as well as incomplete case testing. To address the time lag problem, we showed that two methods provided accurate estimates of the actual CFR_crude_ for COVID-19 even early in the pandemic when the reported CFR_crude_(t) was as much as 10-fold lower than the actual value. The methods were applied to 7 countries with extensive testing. We found by linear regression using the case population age distribution, that the variation in the CFR_crudetimecorrected_ values could be largely explained by three constant age specific CFR coefficients. Therefore, we hypothesized that they provided an accurate estimate of age specific values of CFR_actual_. The hypothesis was validated through comparison with serological testing in NYC, in which the method predicted the percent of the infected adult population more accurately than conventional methods [2, 4, 6, 13, 19, 21–24, 32–34, 41], as well as IFR calculations performed for New York City and other regions well after their initial COVID-19 surges had subsided.

To further assess the accuracy of the calculated CFR_actual_ coefficients we compared them with two later studies which determined age specific IFR coefficients for NYC [25] and from a serological studies performed mainly in Europe in mid-May through early June [44] (Table 4). The Yang et al. study for NYC used a combination of advanced methods to correct for missed cases and extensive access to a wide range of data [25]. The Seoane study used data from large serological studies in multiple countries and corrected them for COVID deaths not included in government reported data [44]. As shown in Table 4, the age specific IFR coefficients they calculated are in excellent agreement with our findings after correction for asymptomatic cases.

**Table 4:** Comparison of age specific IFR coefficients from the present study with serological testing studies internationally [44] and a comprehensive analysis of results from NYC [25] To facilitate comparison, we calculated a 0–64 group mean value for Yang et al. [25] and a 0–69 mean value for Seoane [44] by averaging their reported age sub group IFR values and weighting by percentage of each subgroup of the total infected population.

Yang et al. 2020			Rothman et al. 2021			Seoane 2020		
Age	Age Specific IFR	95% CI	Age	Age Specific IFR	95% CI	Age	Age Specific IFR	95% CI
**0–64**	0.22%		0–69	0.25%	0.09%-0.4%	0–69	0.21%	
**65–74**	4.67%	3.2%-6.7%	70–79	4.70%	2.5%-6.8%	70–79	3.47%	2.9%-4.7%
**75+**	13.88%	9.7%-17.8%	80+	13.82%	10.85%-16.8%	80–90	12.70%	11%-17%

There are several limitations to our study. We did not factor in preexisting conditions which has been reported as significantly affecting mortality [14, 17, 28, 38, 39, 42]. In addition, the derived age group specific CFR_actual_ values may not apply to regions without advanced health care systems. However, even for medically underserved regions, our findings show that targeted high levels of testing in representative local regions could be used to rapidly determine an accurate estimate of CFR_actual_. Another limitation is the need for a rapid determination of the time to fatality distribution function. However, based on our simulations, the early determinations in China were sufficient to obtain accurate CFR_crudetimecorrected_ values. In addition, the closed case method does not depend upon knowing the time to fatality distribution function.

To calculate the IFR from CFR_actual_, we divided the calculated CFR_actual_ by a factor of 2 (50% asymptomatic) from early studies using data from the Diamond Princess [24] and Iceland [11]. This value may be an overestimate, as shown by Mizumoto, because these reports did not fully take into account the lag between infection and the onset of symptoms [34]. However the 50% asymptomatic estimate is still well within the present range of published values, as summarized by the latest CDC update for their best estimate values for the United States [40].

A potential confound in applying our analysis to estimate the percentage of the population infected in a region with limited testing is that the time to death correction, by both methods, assumes a constant fraction of positive case ascertainment. For NYC the validity of this assumption was supported by data from the New York City Department of Health that the number of tests per day was close to constant during the period up to April 22, 2020 and furthermore the total number of deaths reported by mid-June, at which point there would be few remaining fatalities, was similar to our projection based on time correction [17, 38, 39].

As shown in Fig 4, our calculation of the minimum and maximum percentage of the adult population in New York City that has been infected by COVID-19 agreed with the recent studies that performed random testing of segments of the adult population (Fig 4) [28, 42, 43]. In one study, 15.3% of women entering two New York City hospitals to give birth were found by testing to be infected with COVID-19 (33 out of 215 having the virus) [43]. In the second study the New York City infected population was estimated at 21%, this from 3000 serological antibody-based measurements of passersby at testing stations near public areas in New York City and other regions in New York State (with the results reported on April 22, 2020) [42]. The New York City findings were replicated from subsequent testing of 5500 cases reported April 28, 2020 (24% infected) and 15,500 cases reported on May 2, 2020 (19.9% infected). Due to the heterogeneity in COVID-19 fatalities and cases within even New York City, and due to the restricted age range of the groups examined (18–75 for the New York State study), these percent infection values may be overestimates [17, 38]. However, given that the large majority of cases in New York City are between ages 18 and 75, it is unlikely that this bias would have a large impact.

A limitation in determining IFR with serological studies is the percentage of false positives and negatives, which particularly impacts the accuracy when they are applied to region with a low percentage of infections in the population. Since the initial application of serological testing the problem false positives and negatives and how they vary between available tests has been evaluated in detail [45]. The impact of false positives was likely less significant for the New York State study because of the much high percentage of the New York City population that was infected. Additional validation of the New York results is from their finding consistently of low infection percentages (~ 1.0%) in several regions in New York State outside of the New York City metro area which supports a relatively low false positive rate in their testing [17, 28, 42]. Similarly, the serological studies from Europe were from populations with an infection percentage at least several fold higher than anticipated false positives [44].

Having early accurate age specific values CFR_actual_ and IFR is vitally important for predicting the total number of cases and fatalities from COVID-19 and the impact of potential public health measures. As shown in Table 1 and Fig 4, the IFR/CFR_actual_ values used in most of the leading epidemiological models in early May 2020 were not compatible with the number of infections in New York City, and this may have impacted the accuracy of projections of cases and fatalities made at that time. Our approach, in combination with targeted high testing in selected regions, has the potential to accurately determine CFR_actual_ even when adequate testing is not available for the whole population.

## Supporting information

S1 Fig Plots of reported CFR_crude_(t) and closed case CFR_crude_(t) for Australia, Austria, Iceland, Israel, New Zealand, and South Korea. Shown below are plots of the reported closed case CFR_crude_(t) curve and reported CFR_crude_(t) curve for Austria, Australia, Iceland, Israel, New Zealand, and South Korea. The dashed gray line is the value which the closed case CFR(t) has converged to. As for Germany (Fig 1), it is seen that the reported closed case CFR(t) curve converges to a near constant value before the CFR_crude_(t) curve. We found (Fig 2, S2 Fig), that for all countries we examined that the converged value of the closed case CFR was close to the optimum for predicting the CFR_crude_(t) curve, consistent with it being a good approximation of the true corrected CFR for each country.
(PDF)

S2 Fig Plots of simulated and reported N_D_(t) and CFR_crude_(t) curves for Australia, Austria, Iceland, Israel, New Zealand, and South Korea. Similar plots are presented as for Fig 2 for Germany showing the CFR_crude_(t) versus day curves for different values of the corrected CFR. In all cases a lognormal *f*_D_ was used with a median value of 14 days and a logSD of 0.50. The simulated curves calculated using the closed case CFR_crude_ on May 7, 2020 as the corrected CFR value are designated by an asterisk.
(PDF)

S3 Fig Sensitivity analysis to assess the effect of parameters of the lognormal distribution functions. The plots below show results from the sensitivity analysis to assess the effect of the parameters of the lognormal distribution functions (f_D_) on the simulated curves. The approximate best fit value of the corrected CFR was 5.0 (blue line asterisk) which was also the closed case CFR value on the last day plotted. The data from Germany was used for this optimization due to it having the largest number of cases of the nations studied and therefore least susceptible to statistical fluctuations. Fig 1 shows the simulated curves generated for medians of 14, 17, and 21 days and a logSD = 0.50. The effect of increasing the median resulted in the shape of the simulated curves undershooting the reported CFR_crude_(t) curve especially early in the time course due to more deaths being shifted to later dates. Decreasing the median (not shown) had the opposite effect with the simulated curves overshooting the reported data early in the time course. We also examined the effect of the logSD value on the simulated curves. Fig 2 shows the simulated curves generated for a median of 14 days and logSD values of 0.25, 0.5, and 0.75. The sensitivity logSD throughout that range was found to be low with an optimum at 0.50 which is consistent with the original reports [1, 2].
(PDF)

S4 Fig Simulated closed case CFR curves for Germany and South Korea. In order to understand the basis of the early convergence of the closed case CFR we performed simulations of its time course using cases per day of from Germany and South Korea. Less information is available about the recovery distribution function than the fatality distribution function (*f*_R_). Based on the study of SARS by Ghani and coworkers *f*_R_ is substantially less skewed than *F*_D_ [1]. This finding is consistent with the reports from early data obtained in China for COVID-19 by Bi et al. and Verity et al. who also found that the median of the *f*_R_ was several days later than for *f*_D_ [2, 3]. We assessed the impact of the time to recovery distribution function by simulated the closed case CFR curve using the optimum *f*_R_ (median 14 days, logSD 0.50) to calculate N_D_(t) and *f*_R_ distributions with logSD = 0.25 and median values of 14 days, 16 days, and 18 days. For input data we used the number cases per day for Germany and South Korea. The corrected CFR for each country was used in the simulations. Below we show the simulated closed case CFR curves for Germany and South Korea. Also plotted is the simulated crude CFR curve for each country. It is seen that for all of the recovery distributions evaluated the closed case CFR initially overshoots the corrected CFR value and then converges to it. The smallest overshoot and fastest convergence was for when *f*_R_ had the same median value as *f*_D_. In all cases the CFR_crude_ curve took longer to converge than the closed case CFR curve, consistent with the reported data from Germany and South Korea (Fig 1 and S1 Fig). The decay portion of the closed case CFR curve for South Korea was consistent with a *f*_R_ median of 16 days while for Germany a 14-day median better predicted the rapid convergence to the corrected CFR values. The reported initial rise in the closed case CFR for both countries was less well predicted by the simulations, potentially due to differences in the criteria for recovery early in the outbreaks.
(PDF)

S1 Table Ratio of total to positive tests and tests per 1,000,000 in the population. This table shows the ratio of negative COVID-19 tests to 1 positive COVID-19 test, and the number of COVID-19 tests per 1,000,000 in the population for each of the 7 countries included in our analysis, as of May 10, 2020 [1–8].
(PDF)
