# Supplementary material for: Analysis of the time course of COVID-19 cases and deaths from countries with extensive testing allows accurate early estimates of the age specific symptomatic CFR values
Source: PLoS One. 2021 Aug 18;16(8):e0253843. doi: 10.1371/journal.pone.0253843 (PMC8372929; doi:10.1371/journal.pone.0253843)

S 1 Fig. Plots of reported $CFR_{crude}(t)$ and closed case $CFR_{crude}(t)$ for Australia, Austria, Iceland, Israel, New Zealand, and South Korea.

Shown below are plots of the reported closed case $CFR_{crude}(t)$ curve and reported $CFR_{crude}(t)$ curve for Austria, Australia, Iceland, Israel, New Zealand, and South Korea. The dashed gray line is the value which the closed case CFR(t) has converged to. As for Germany (Figure 4), it is seen that the reported closed case CFR(t) curve converges to a near constant value before the $CFR_{crude}(t)$ curve. We found (Figure 5, S 2 Fig.), that for all countries we examined that the converged value of the closed case CFR was close to the optimum for predicting the $CFR_{crude}(t)$ curve, consistent with it being a good approximation of the true corrected CFR for each country.

(A) Australia

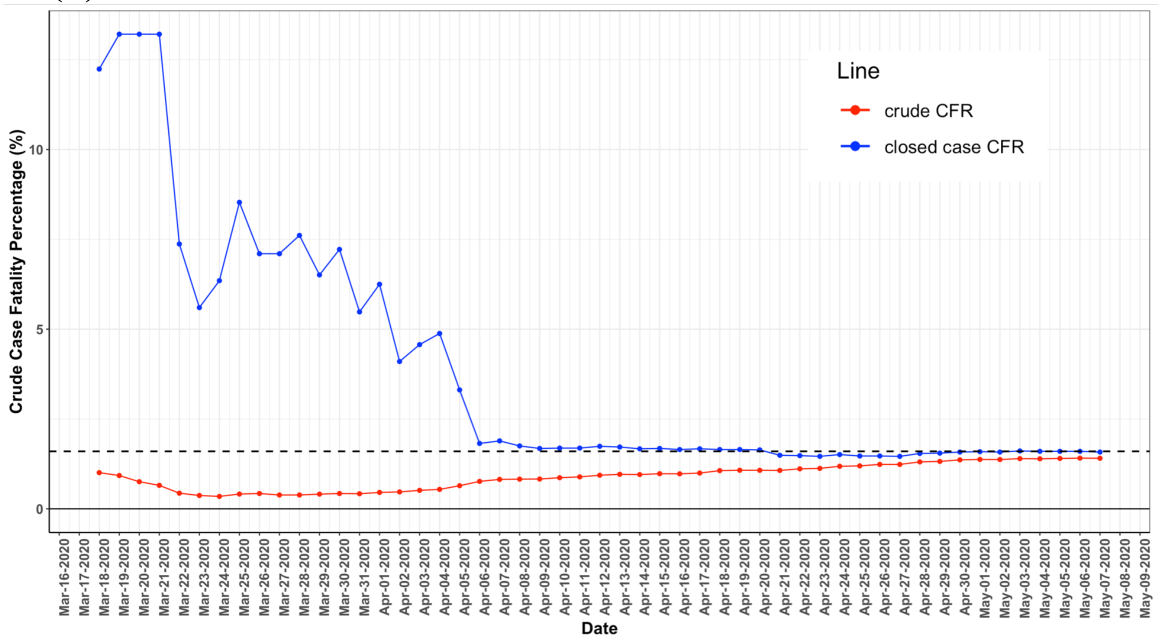

(B) Austria

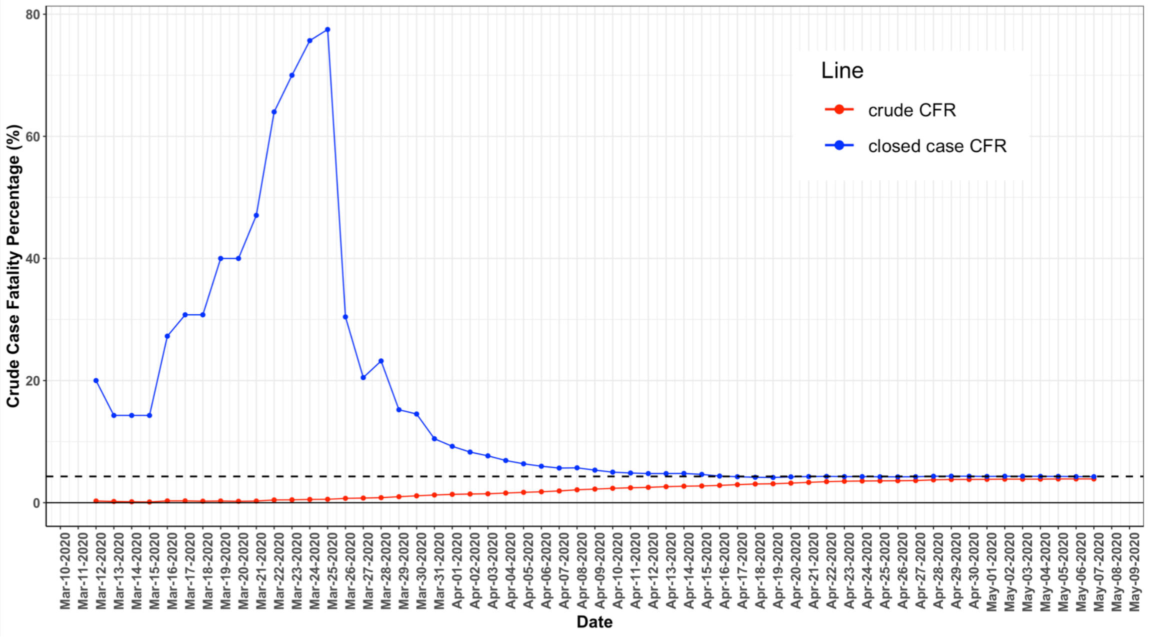

(C) Iceland

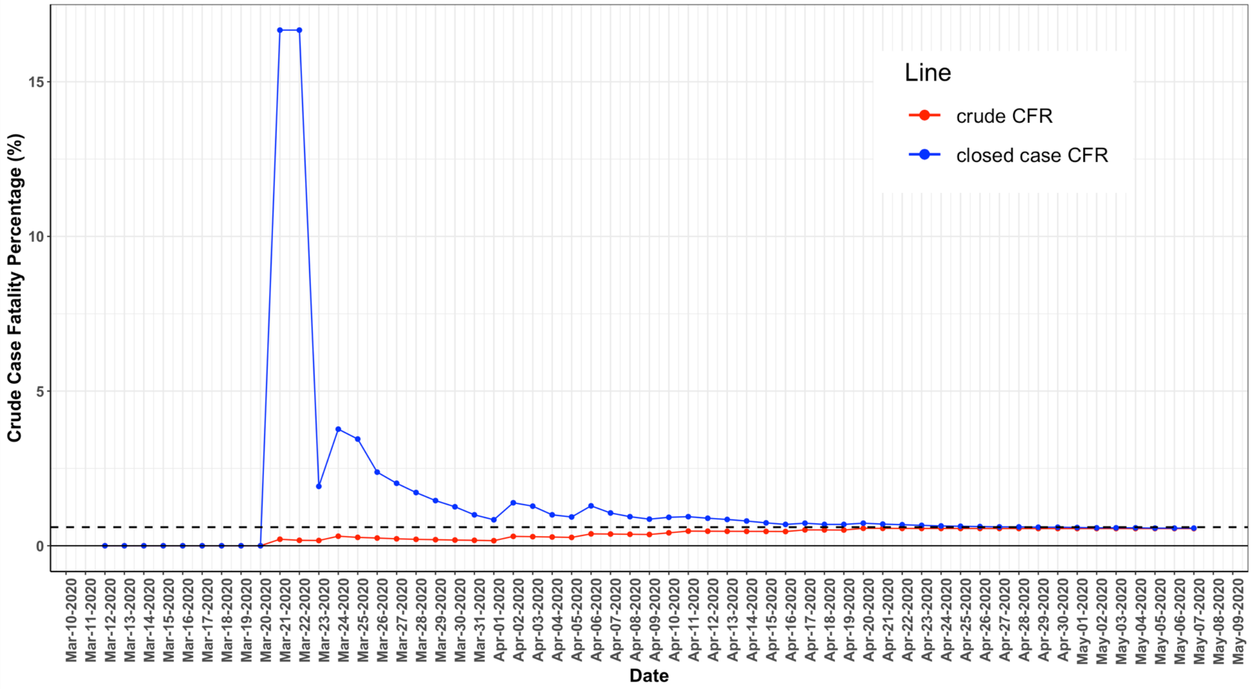

(D) Israel

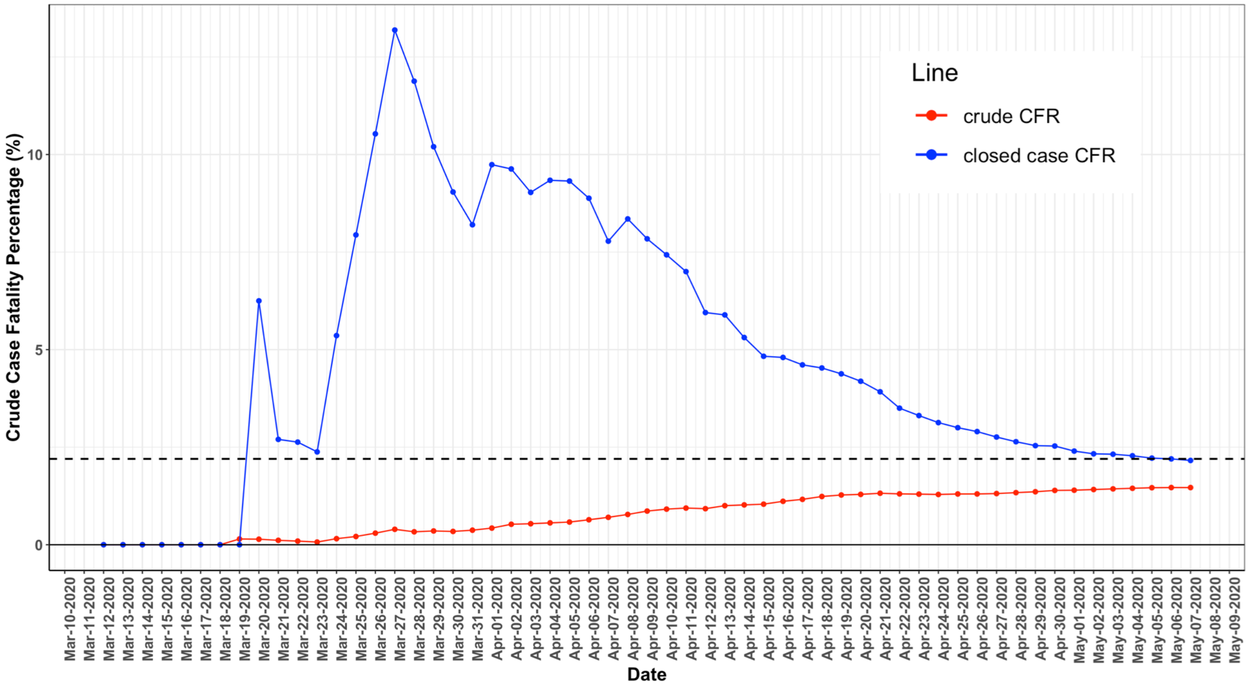

(E) New Zealand

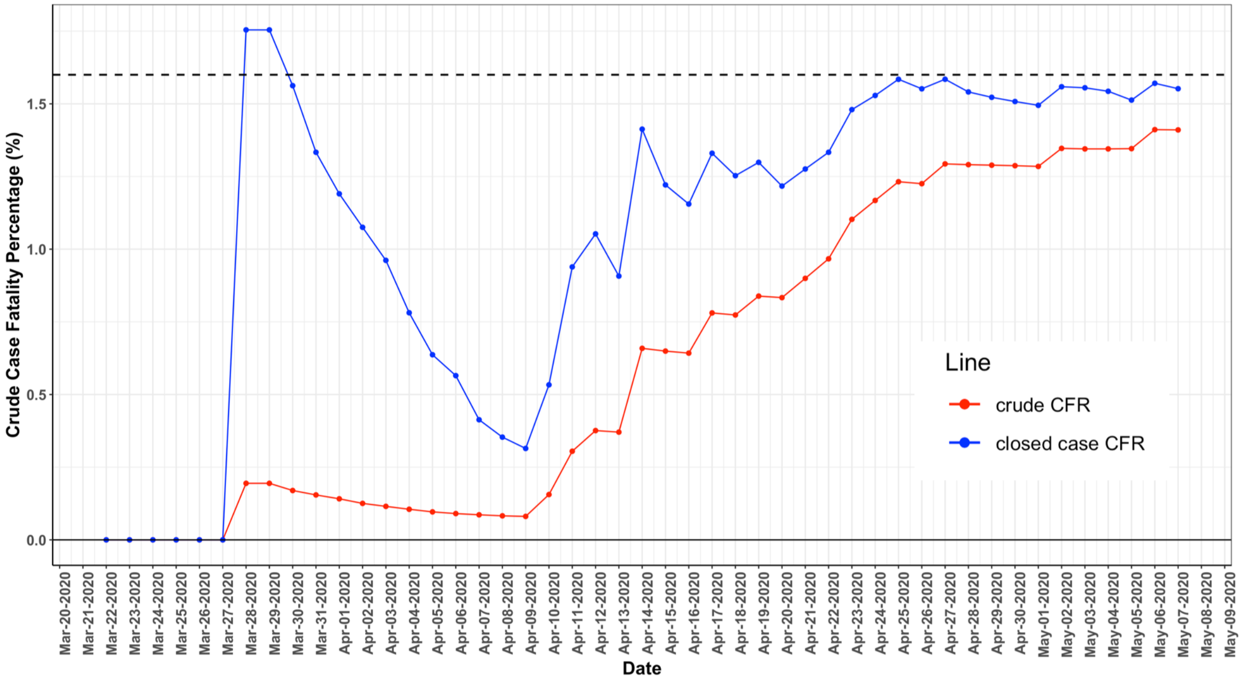

(F) South Korea

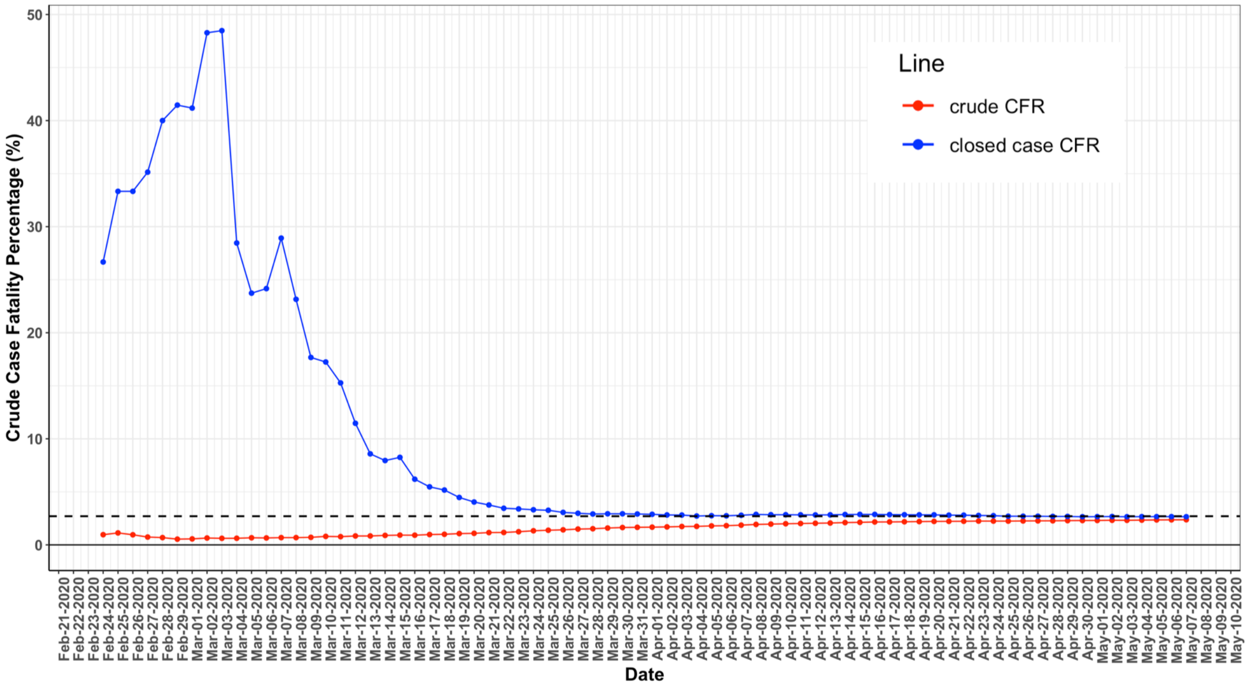

Supplement: S1 Fig — Shown below are plots of the reported closed case CFRcrude(t) curve and reported CFRcrude(t) curve for Austria, Australia, Iceland, Israel, New Zealand, and South Korea. The dashed gray line is the value which the closed case CFR(t) has converged to. As for Germany (Fig 1), it is seen that the reported closed case CFR(t) curve converges to a near constant value before the CFRcrude(t) curve. We found (Fig 2, S2 Fig), that for all countries we examined that the converged value of the closed case CFR was close to the optimum for predicting the CFRcrude(t) curve, consistent with it being a good approximation of the true corrected CFR for each country. (PDF) [file pone.0253843.s001.pdf]
