# Supplementary material for: Analysis of the time course of COVID-19 cases and deaths from countries with extensive testing allows accurate early estimates of the age specific symptomatic CFR values
Source: PLoS One. 2021 Aug 18;16(8):e0253843. doi: 10.1371/journal.pone.0253843 (PMC8372929; doi:10.1371/journal.pone.0253843)
Supplement: S2 Fig — Similar plots are presented as for Fig 2 for Germany showing the CFRcrude(t) versus day curves for different values of the corrected CFR. In all cases a lognormal fD was used with a median value of 14 days and a logSD of 0.50. The simulated curves calculated using the closed case CFRcrude on May 7, 2020 as the corrected CFR value are designated by an asterisk. (PDF) [file pone.0253843.s002.pdf]

S 2 Fig. Plots of simulated and reported $N_D(t)$ and $CFR_{crude}(t)$ curves for Australia, Austria, Iceland, Israel, New Zealand, and South Korea.

Similar plots are presented as for Figures 5A and 5B for Germany showing the $CFR_{crude}(t)$ versus day curves for different values of the corrected CFR. In all cases a lognormal f_D was used with a median value of 14 days and a logSD of 0.50. The simulated curves calculated using the closed case CFR_{crude} on May 7, 2020 as the corrected CFR value are designated by an asterisk.

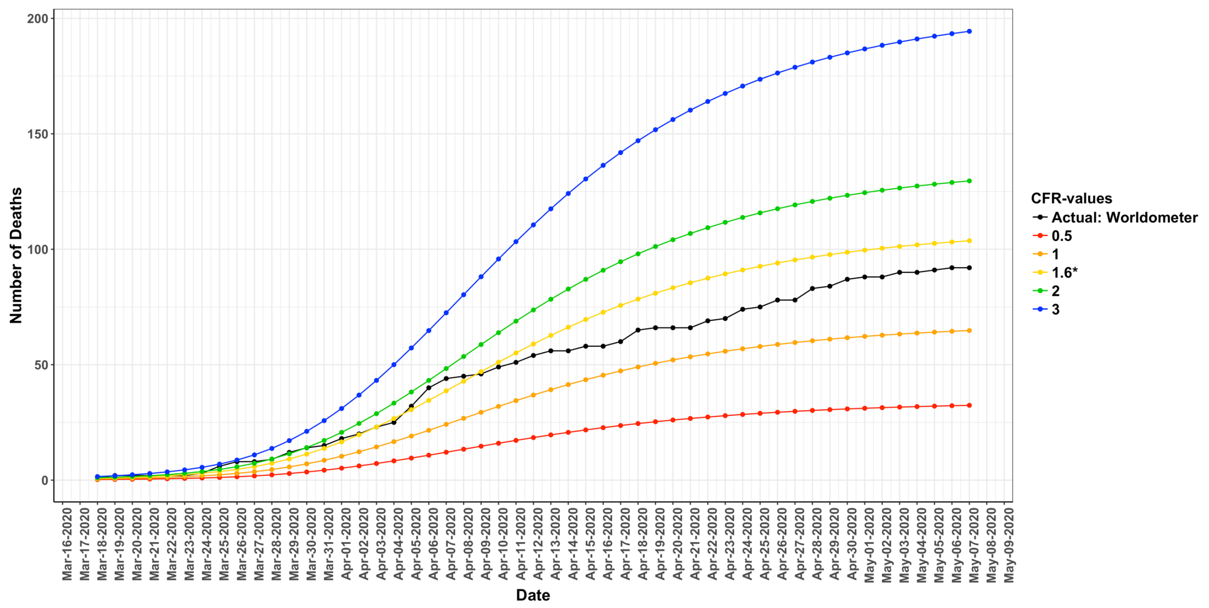

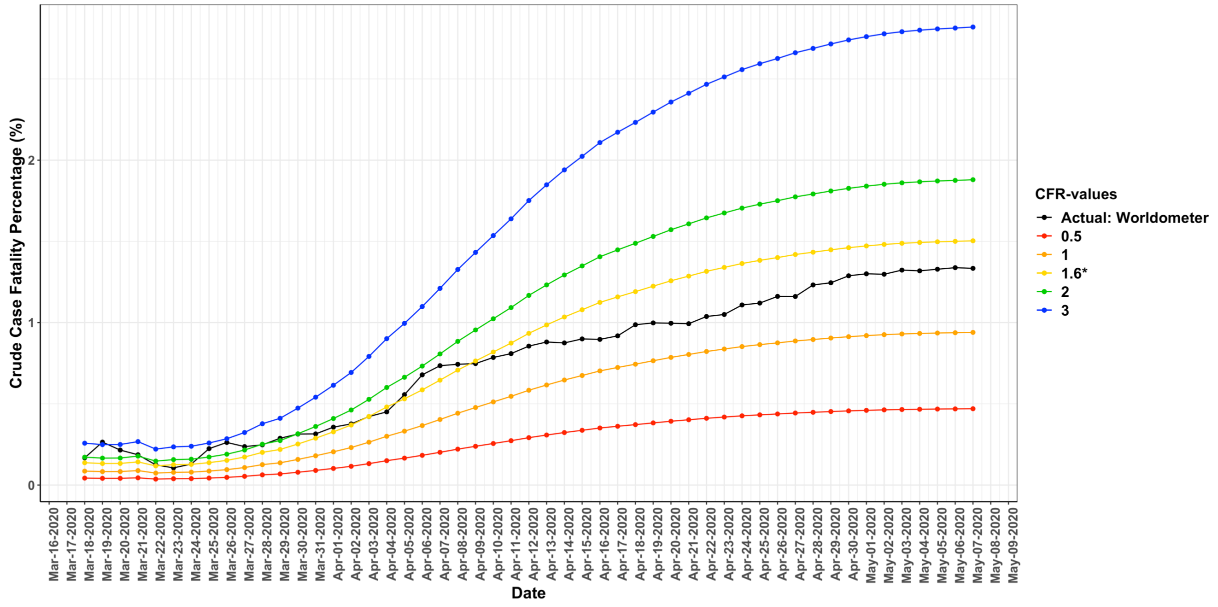

(A). Simulated $CFR_{crude}(t)$ and $N_D(t)$ curves for Australia. The simulated curves shown were calculated using a corrected CFR of 0.50, 1.0, 1.7 (the closed case CFR_{crude}), 2.0, and 3.0. The reported data is shown in black.

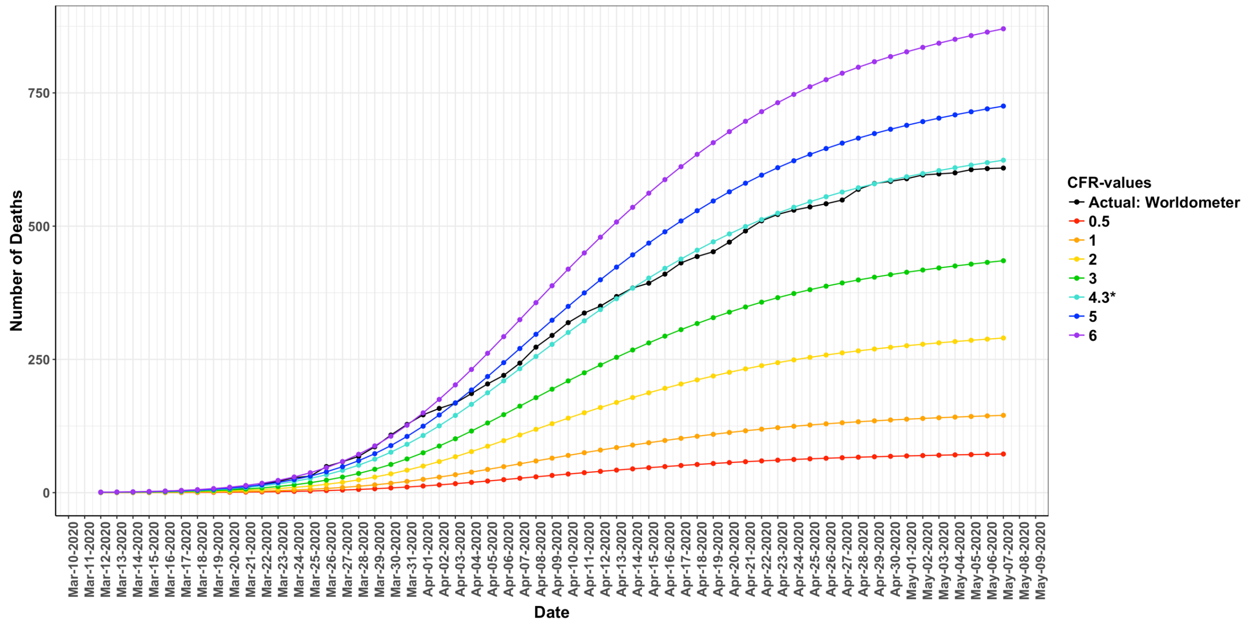

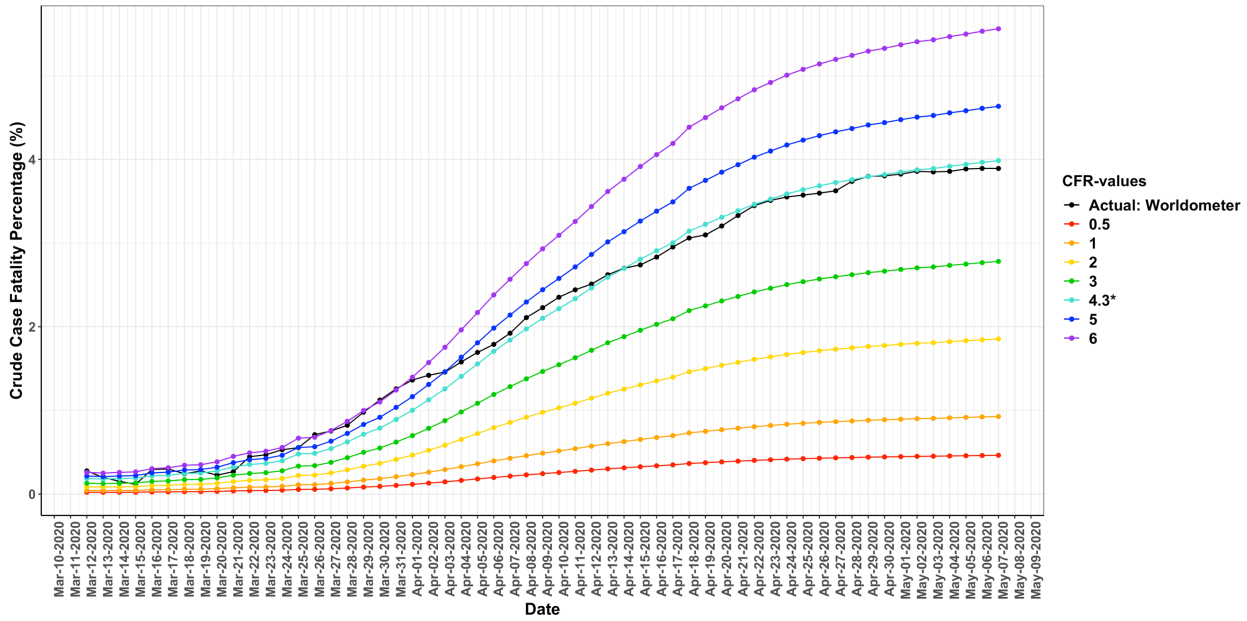

(B). Simulated $CFR_{crude}(t)$ and $N_D(t)$ curves for Austria. The simulated curves shown were calculated using a corrected CFR of 0.50, 1.0, 2.0, 3.0, 4.3(the closed case CFR_{crude}), 5.0, and 6.0. The reported data is shown in black.

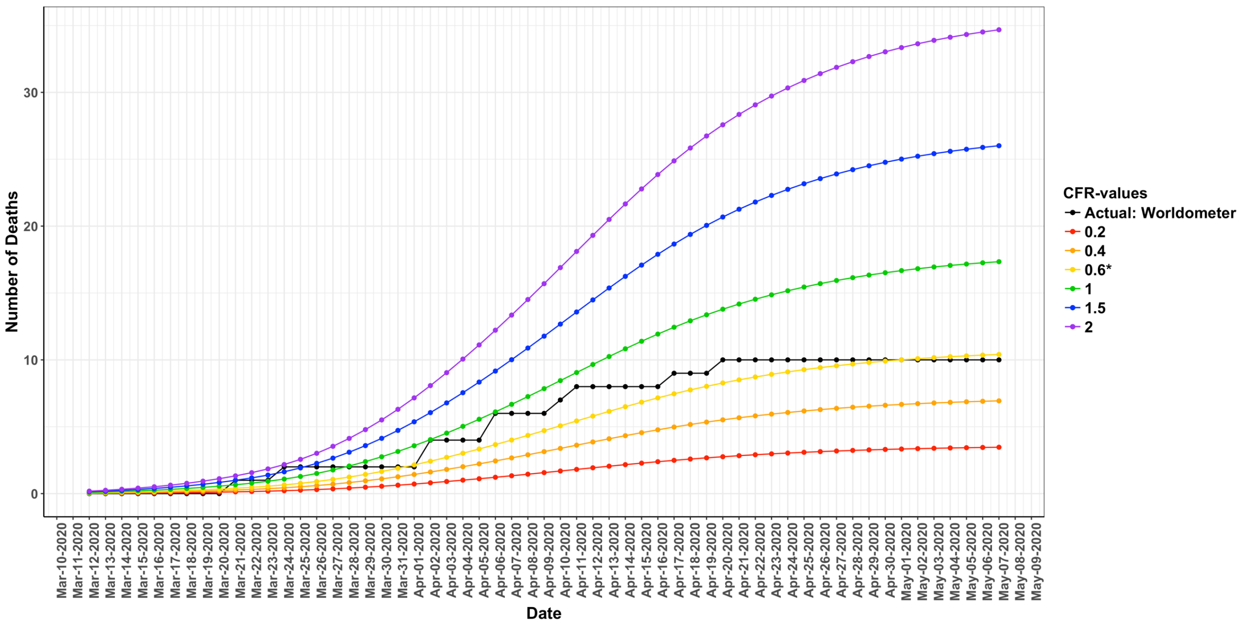

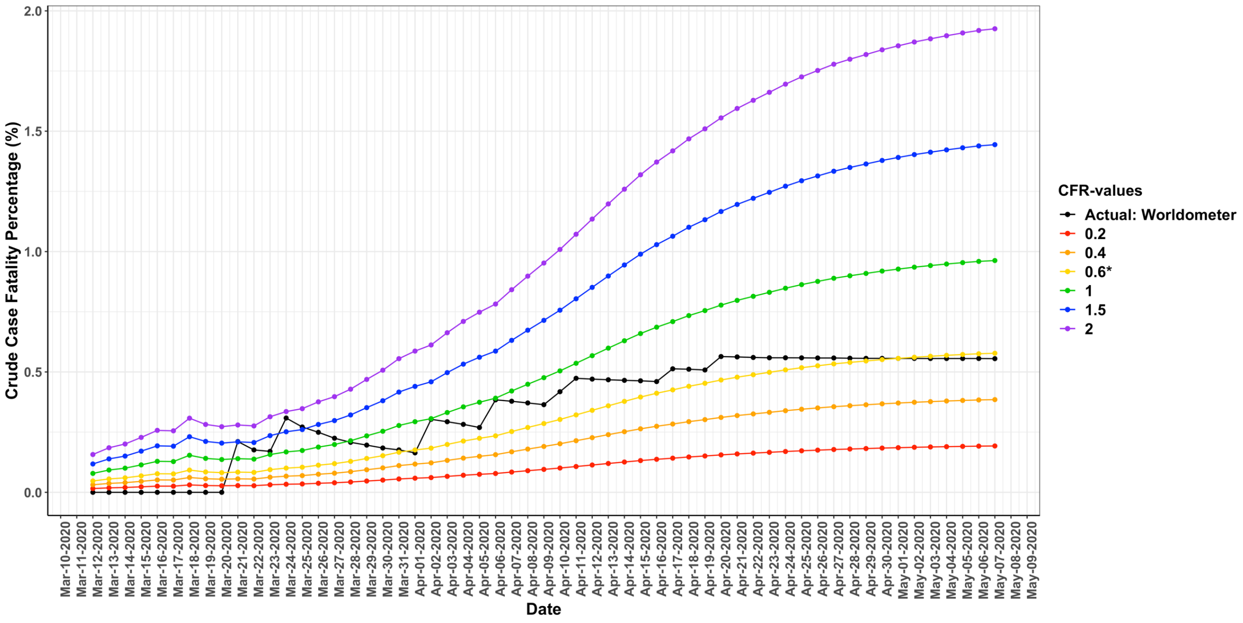

(C). Simulated $CFR_{crude}(t)$ and $N_D(t)$ curves for Iceland. The simulated curves shown were calculated using a corrected CFR of 0.20, 0.50, 0.70 (the closed case CFR_{crude}), 1.0, 1.5, and 2.0. The reported data is shown in black.

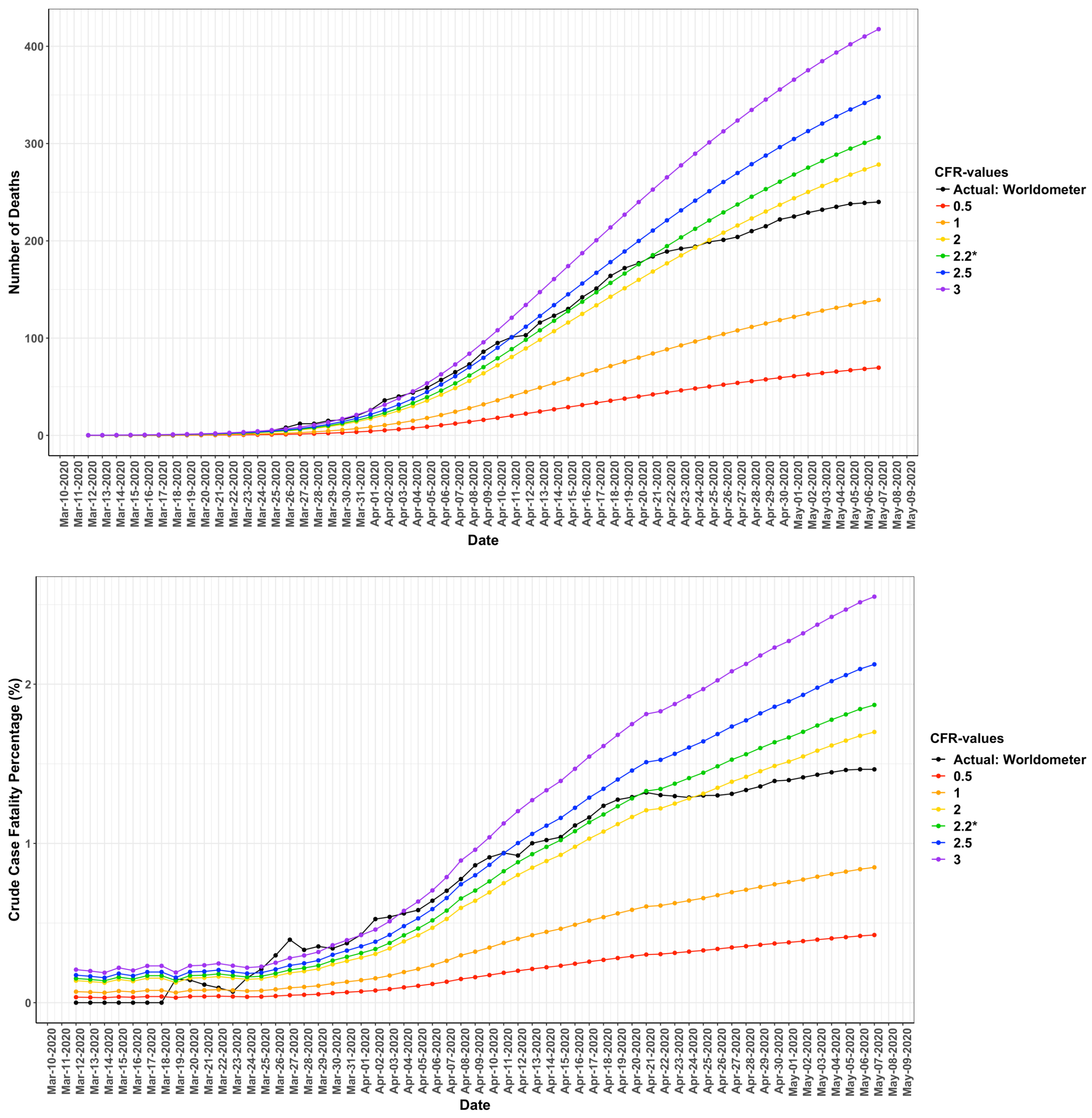

(D). Simulated $CFR_{crude}(t)$ and $N_D(t)$ curves for Israel. The simulated curves shown were calculated using a corrected CFR of 0.50, 1.0, 2.0, 2.2 (the closed case CFR_{crude}), 2.5, and 3.0. The reported data is shown in black.

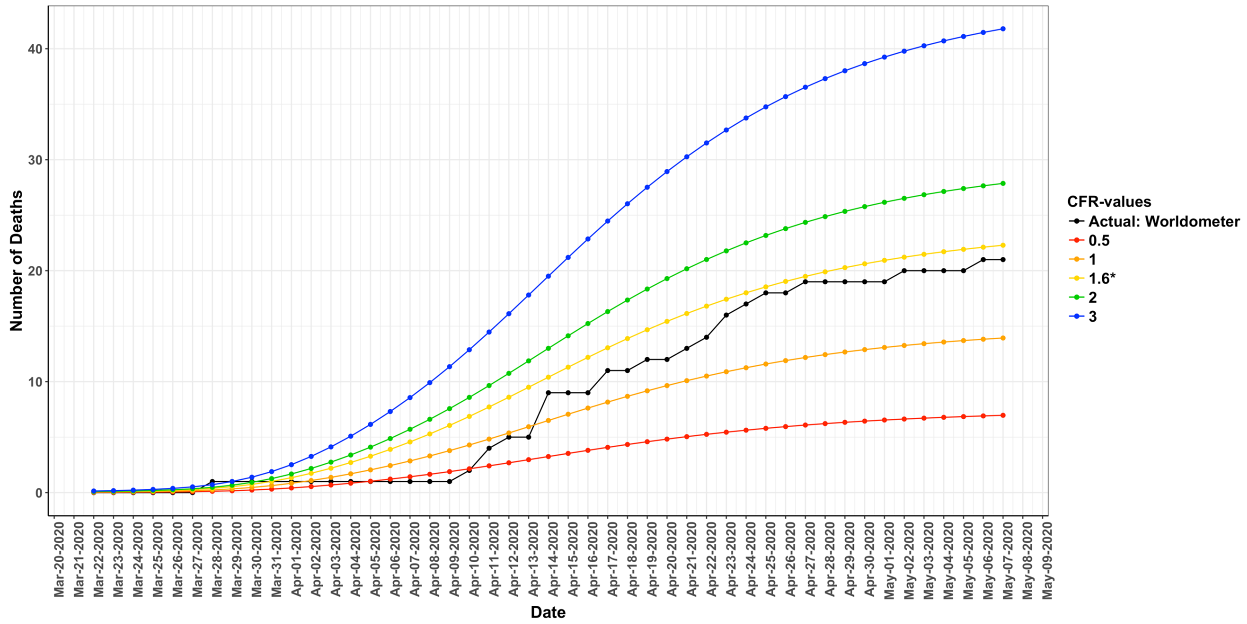

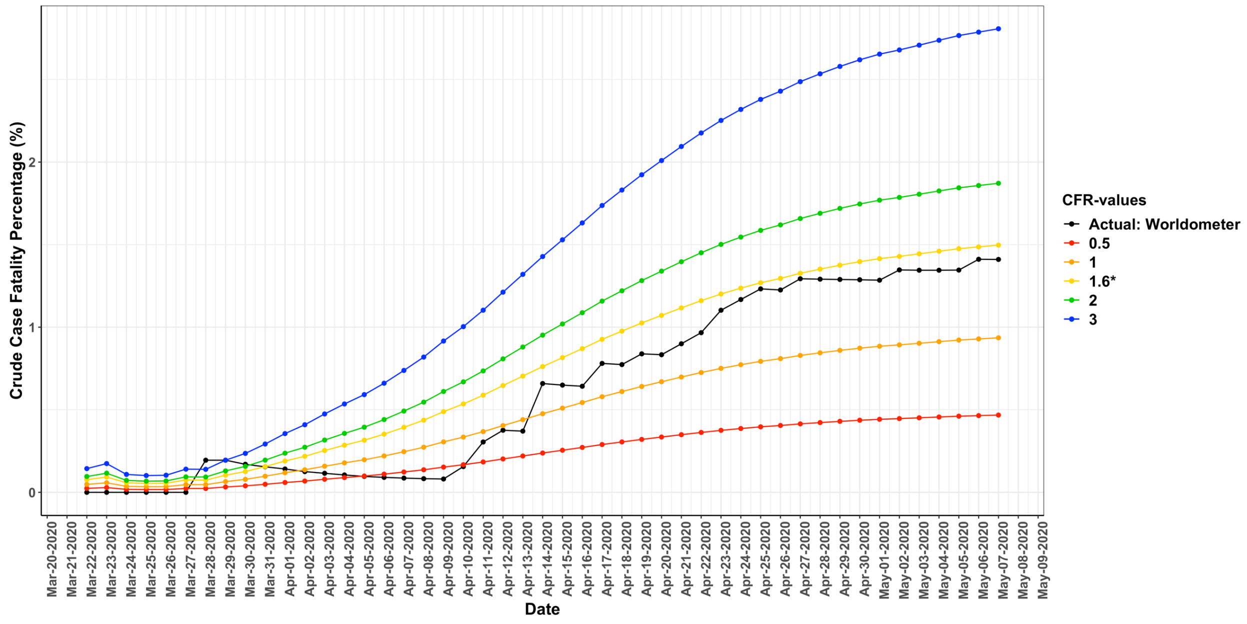

(E). Simulated $CFR_{crude}(t)$ and $N_D(t)$ curves for New Zealand. The simulated curves shown were calculated using a corrected CFR of 0.50, 1.0, 1.6 (the closed case CFR_{crude}), 2.0, and 3.0. The reported data is shown in black.

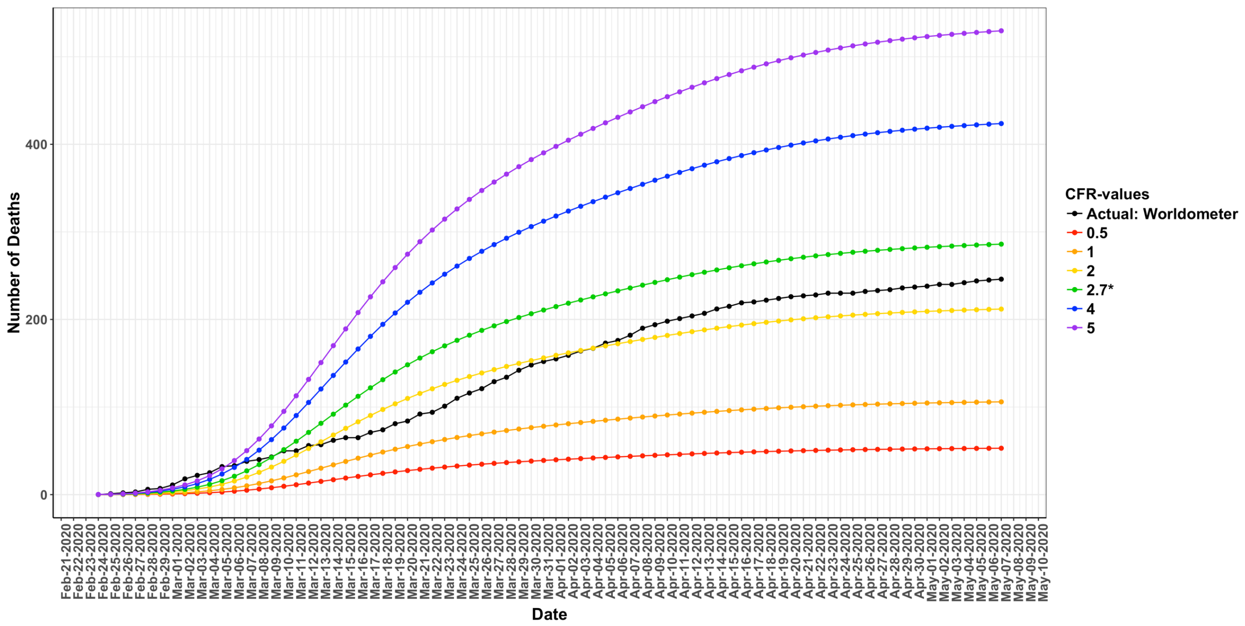

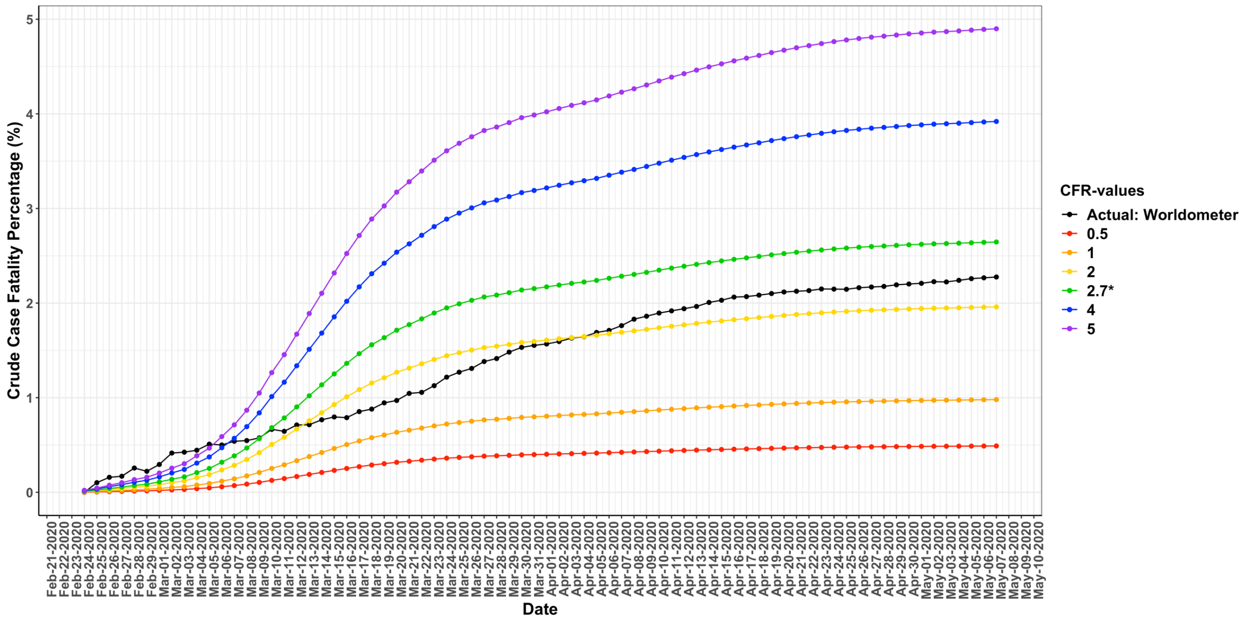

(F). Simulated $CFR_{crude}(t)$ and $N_D(t)$ curves for South Korea. The simulated curves shown were calculated using a corrected CFR of 0.50, 1.0, 2.0, 2.5 (the closed case CFR_{crude}), 4.0, and 5.0. The reported data is shown in black.
