# Supplementary material for: Analysis of the time course of COVID-19 cases and deaths from countries with extensive testing allows accurate early estimates of the age specific symptomatic CFR values
Source: PLoS One. 2021 Aug 18;16(8):e0253843. doi: 10.1371/journal.pone.0253843 (PMC8372929; doi:10.1371/journal.pone.0253843)
Supplement: S3 Fig — The plots below show results from the sensitivity analysis to assess the effect of the parameters of the lognormal distribution functions (fD) on the simulated curves. The approximate best fit value of the corrected CFR was 5.0 (blue line asterisk) which was also the closed case CFR value on the last day plotted. The data from Germany was used for this optimization due to it having the largest number of cases of the nations studied and therefore least susceptible to statistical fluctuations. Fig 1 shows the simulated curves generated for medians of 14, 17, and 21 days and a logSD = 0.50. The effect of increasing the median resulted in the shape of the simulated curves undershooting the reported CFRcrude(t) curve especially early in the time course due to more deaths being shifted to later dates. Decreasing the median (not shown) had the opposite effect with the simulated curves overshooting the reported data early in the time course. We also examined the effect of the logSD value on the simulated curves. Fig 2 shows the simulated curves generated for a median of 14 days and logSD values of 0.25, 0.5, and 0.75. The sensitivity logSD throughout that range was found to be low with an optimum at 0.50 which is consistent with the original reports [1, 2]. (PDF) [file pone.0253843.s003.pdf]

S 3 Fig. Sensitivity analysis to assess the effect of parameters of the lognormal distribution functions. The plots below show results from the sensitivity analysis to assess the effect of the parameters of the lognormal distribution functions (f_D) on the simulated curves. The approximate best fit value of the corrected CFR was 5.0 (blue line asterisk) which was also the closed case CFR value on the last day plotted. The data from Germany was used for this optimization due to it having the largest number of cases of the nations studied and therefore least susceptible to statistical fluctuations. Figure A shows the simulated curves generated for medians of 14, 17, and 21 days and a logSD= 0.50. The effect of increasing the median resulted in the shape of the simulated curves undershooting the reported $CFR_{crude}(t)$ curve especially early in the time course due to more deaths being shifted to later dates. Decreasing the median (not shown) had the opposite effect with the simulated curves overshooting the reported data early in the time course.

We also examined the effect of the logSD value on the simulated curves. Figure B shows the simulated curves generated for a median of 14 days and logSD values of 0.25, 0.5, and 0.75. The sensitivity logSD throughout that range was found to be low with an optimum at 0.50 which is consistent with the original reports [1,2].

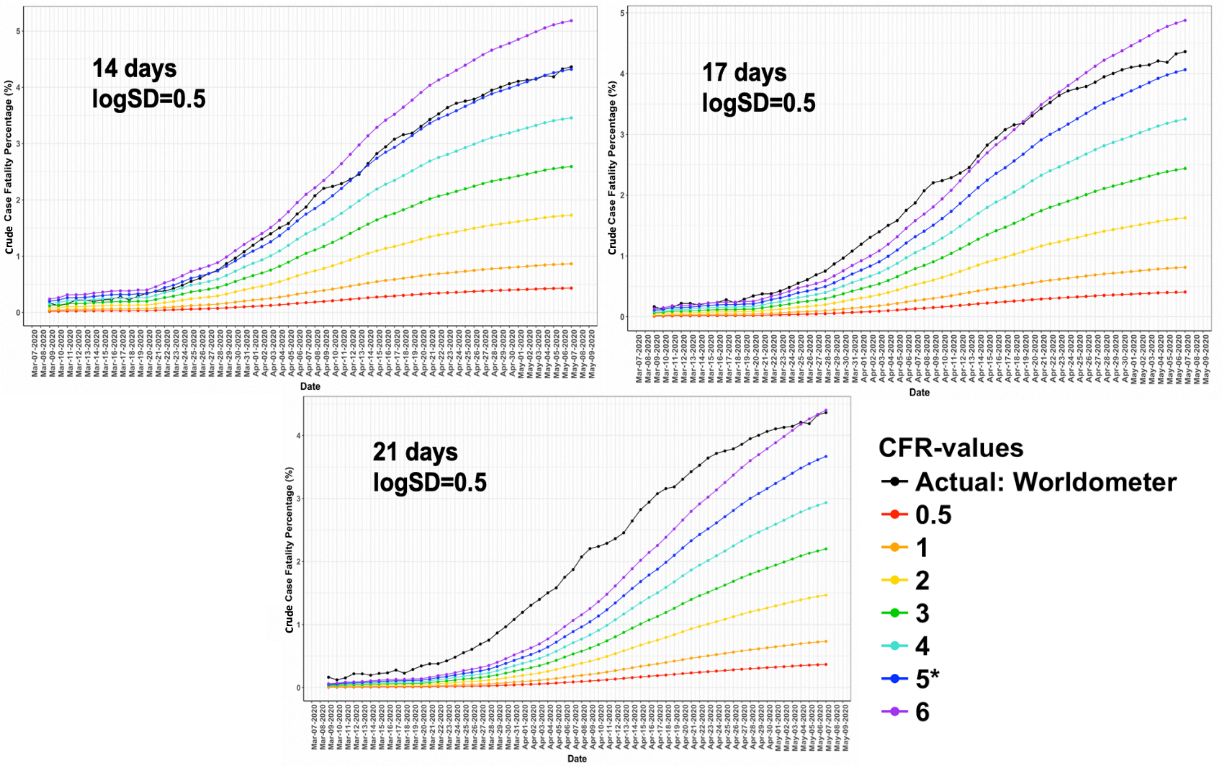

(A). Simulated $CFR_{crude}(t)$ curves for medians of 14 days, 17 days, and 21 days with LogSD = 0.50. The best match to the reported data was for a median of 14 days.

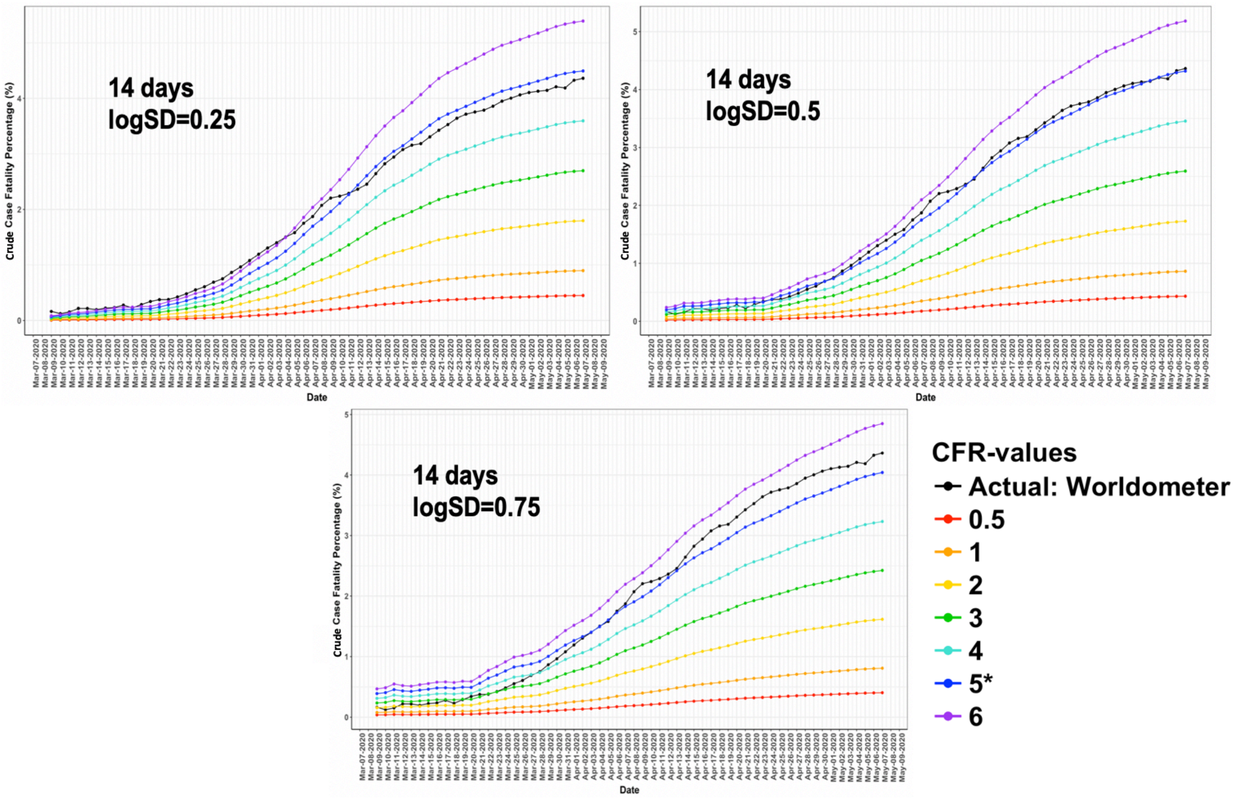

(B). Simulations of $CFR_{crude}(t)$ for a log normal distribution function with medians of 14 days and a logSD of 0.25, 0.50, and 0.75. The best fit of the simulated curves to the reported data is for a logSD of 0.50, however the sensitivity to changes in the log-normal distribution was low. We also examined logSD values of 0.60 and 0.40.

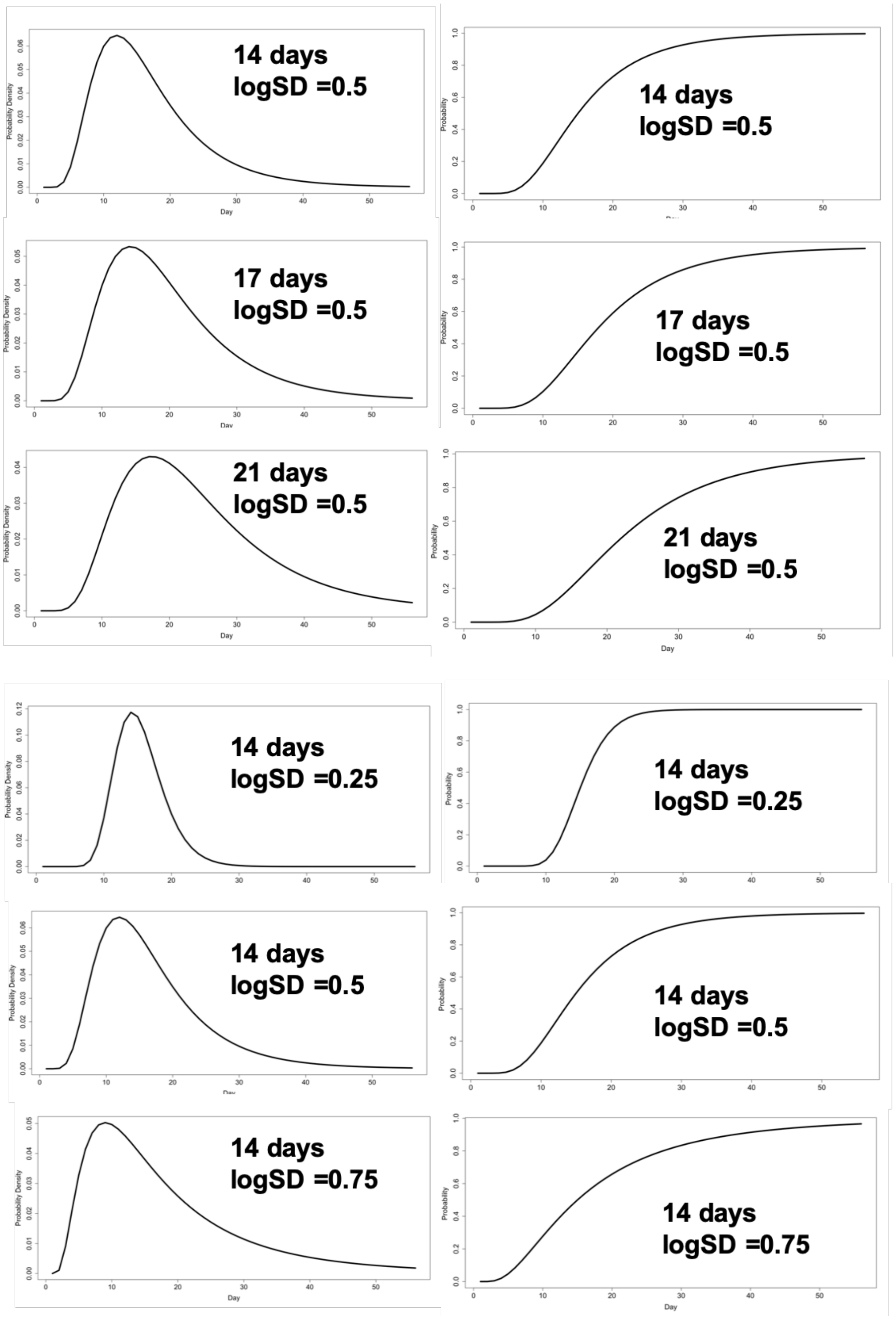

(C). Lognormal probability (f_D) and cumulative (F_D) distribution functions used for the simulations in Figures A and B.

References

1. Mizumoto K, Chowell G. Estimating Risk for Death from 2019 Novel Coronavirus Disease, China, January - February 2020. Emerg Infect Dis. 2020;26(6):1–16.

2. Linton NM, Kobayashi T, Yang Y, Hayashi K, Akhmetzhanov AR, Jung S, et al. Incubation Period and Other Epidemiological Characteristics of 2019 Novel Coronavirus Infections with Right Truncation: A Statistical Analysis of Publicly Available Case Data. J Clin Med. 2020;9(2):538.
