# Supplementary material for: Analysis of the time course of COVID-19 cases and deaths from countries with extensive testing allows accurate early estimates of the age specific symptomatic CFR values
Source: PLoS One. 2021 Aug 18;16(8):e0253843. doi: 10.1371/journal.pone.0253843 (PMC8372929; doi:10.1371/journal.pone.0253843)
Supplement: S4 Fig — In order to understand the basis of the early convergence of the closed case CFR we performed simulations of its time course using cases per day of from Germany and South Korea. Less information is available about the recovery distribution function than the fatality distribution function (fR). Based on the study of SARS by Ghani and coworkers fR is substantially less skewed than FD [1]. This finding is consistent with the reports from early data obtained in China for COVID-19 by Bi et al. and Verity et al. who also found that the median of the fR was several days later than for fD [2, 3]. We assessed the impact of the time to recovery distribution function by simulated the closed case CFR curve using the optimum fR (median 14 days, logSD 0.50) to calculate ND(t) and fR distributions with logSD = 0.25 and median values of 14 days, 16 days, and 18 days. For input data we used the number cases per day for Germany and South Korea. The corrected CFR for each country was used in the simulations. Below we show the simulated closed case CFR curves for Germany and South Korea. Also plotted is the simulated crude CFR curve for each country. It is seen that for all of the recovery distributions evaluated the closed case CFR initially overshoots the corrected CFR value and then converges to it. The smallest overshoot and fastest convergence was for when fR had the same median value as fD. In all cases the CFRcrude curve took longer to converge than the closed case CFR curve, consistent with the reported data from Germany and South Korea (Fig 1 and S1 Fig). The decay portion of the closed case CFR curve for South Korea was consistent with a fR median of 16 days while for Germany a 14-day median better predicted the rapid convergence to the corrected CFR values. The reported initial rise in the closed case CFR for both countries was less well predicted by the simulations, potentially due to differences in the criteria for recovery early in the outbreaks. (PDF) [file pone.0253843.s004.pdf]

S 4 Fig. Simulated closed case CFR curves for Germany and South Korea. In order to understand the basis of the early convergence of the closed case CFR we performed simulations of its time course using cases per day of from Germany and South Korea. Less information is available about the recovery distribution function than the fatality distribution function (f_R). Based on the study of SARS by Ghani and coworkers f_R is substantially less skewed than F_D [1]. This finding is consistent with the reports from early data obtained in China for COVID-19 by Bi et al. and Verity et al. who also found that the median of the f_R was several days later than for f_D [2,3]. We assessed the impact of the time to recovery distribution function by simulated the closed case CFR curve using the optimum f_R (median 14 days, logSD 0.50) to calculate $N_D(t)$ and f_R distributions with logSD = 0.25 and median values of 14 days, 16 days, and 18 days. For input data we used the number cases per day for Germany and South Korea. The corrected CFR for each country was used in the simulations.

Below we show the simulated closed case CFR curves for Germany and South Korea. Also plotted is the simulated crude CFR curve for each country. It is seen that for all of the recovery distributions evaluated the closed case CFR initially overshoots the corrected CFR value and then converges to it. The smallest overshoot and fastest convergence was for when f_R had the same median value as f_D. In all cases the CFR_{crude} curve took longer to converge than the closed case CFR curve, consistent with the reported data from Germany and South Korea (Figure 4 and S 1 Fig.). The decay portion of the closed case CFR curve for South Korea was consistent with a f_R median of 16 days while for Germany a 14-day median better predicted the rapid convergence to the corrected CFR values. The reported initial rise in the closed case CFR for both countries was less well predicted by the simulations, potentially due to differences in the criteria for recovery early in the outbreaks.

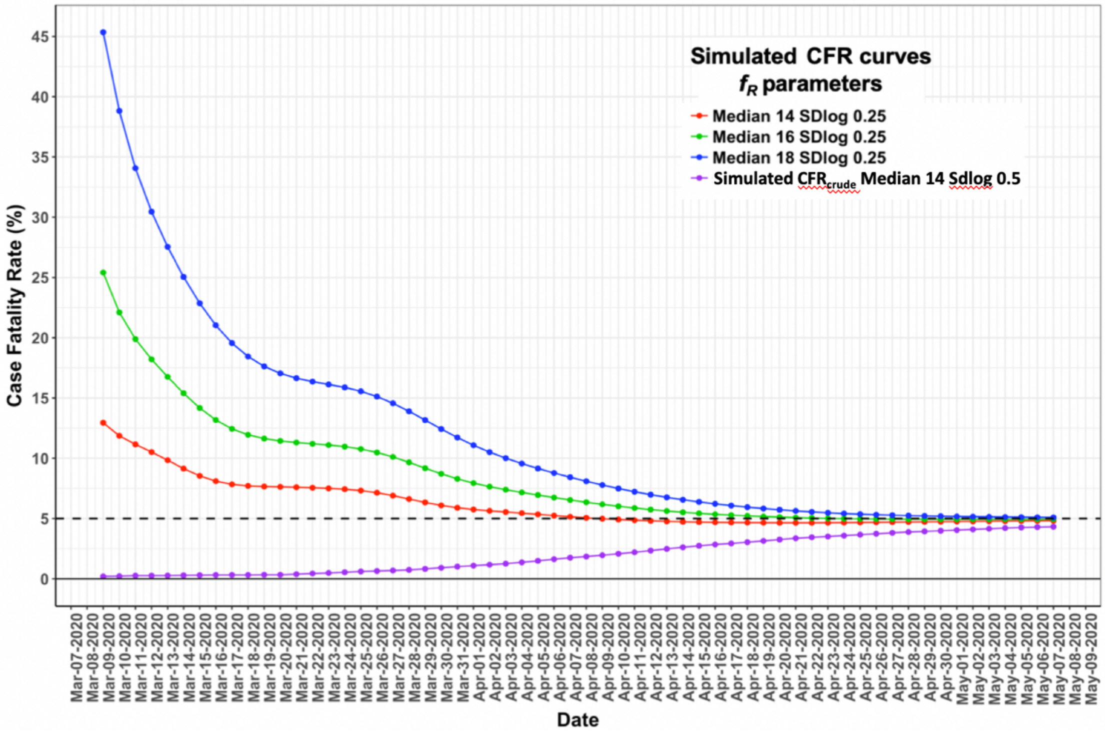

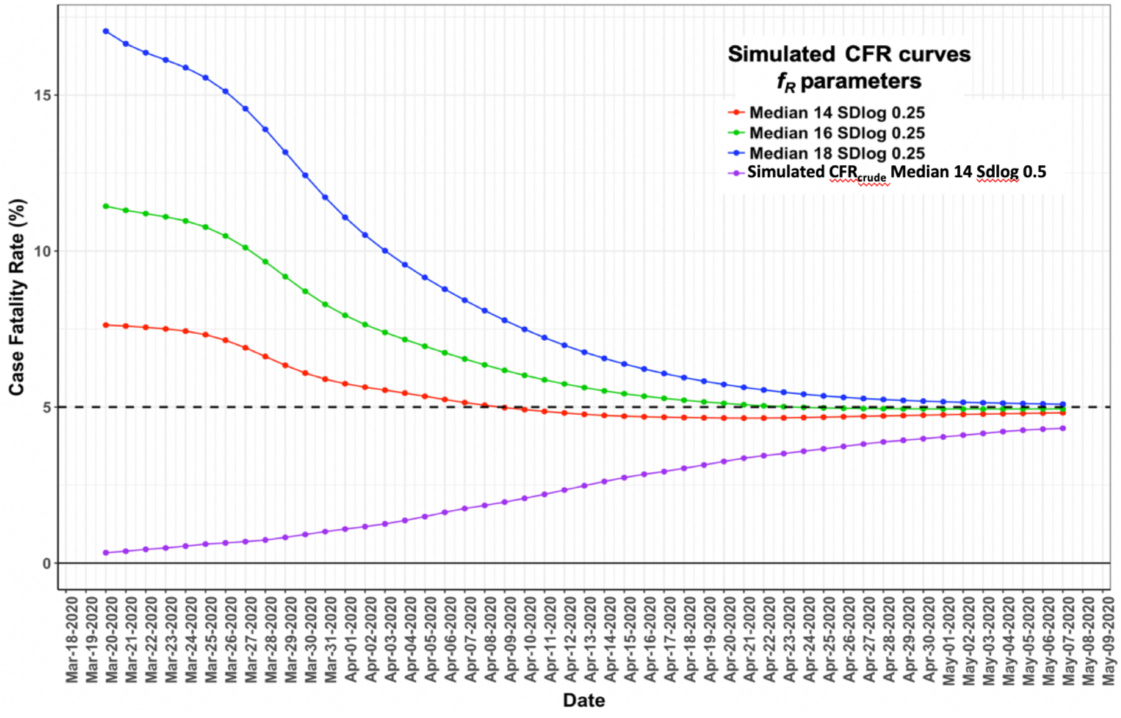

(A). Simulated closed case and crude CFR curves for Germany. 1. Full time course (starting at 10 days after the first 100 cases) 2. Time course starting at March 20, 2020. Curves shown are for time to recovery distribution functions with logSD= 0.25 and medians of 14 (red), 16 (green), and 18 (blue). The CFR_{crude} curve (purple) is also shown. In all cases the closed case CFR is seen to initially rise above the corrected CFR (dashed black line, cCFR = 5.0) and then converge back to it. Convergence was fastest when the median was the same for the time to death and time to recovery distributions.

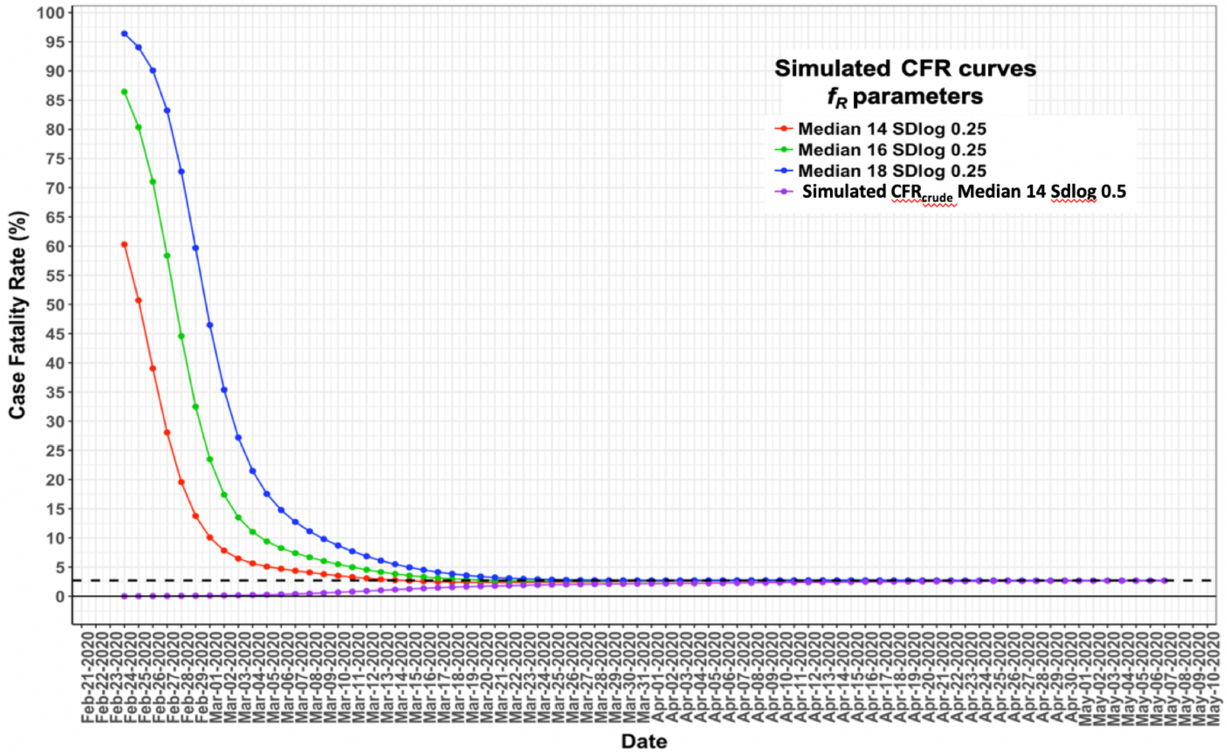

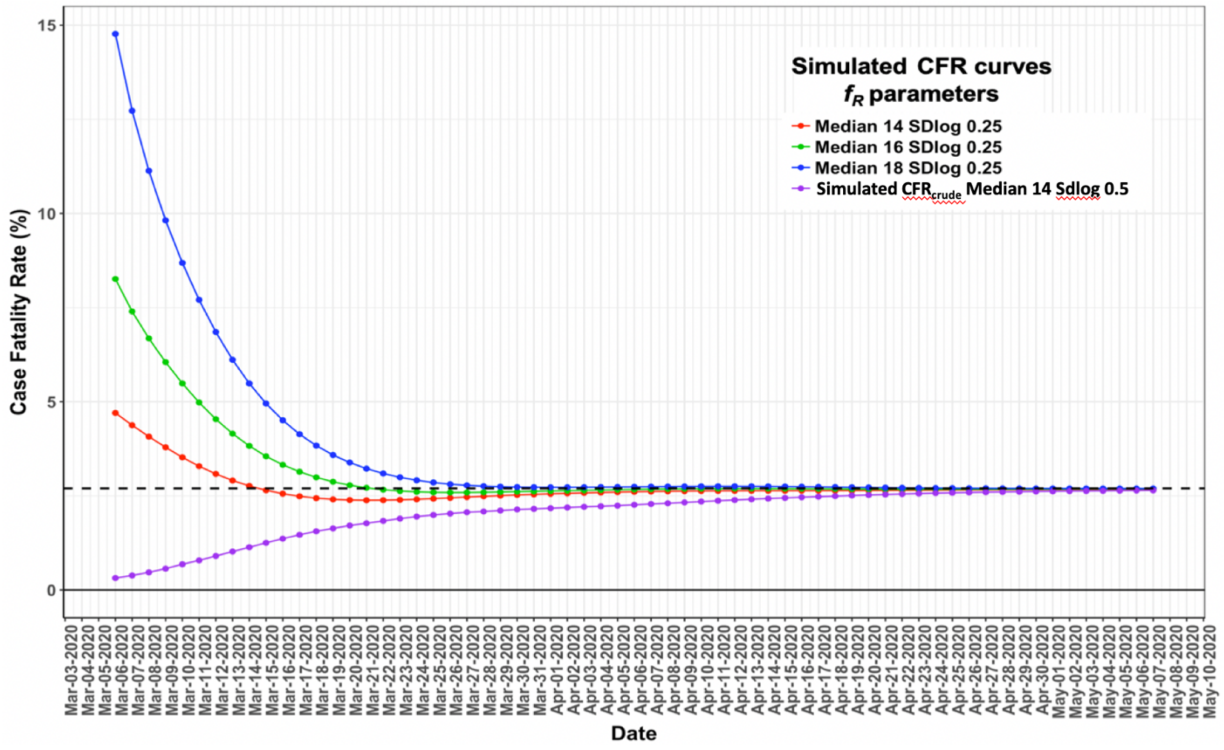

(B). Simulated closed case and crude CFR curves for South Korea. 1. Full time course (starting at 10 days after the first 100 cases). 2. Time course staring at March 5, 2020. Similar behavior is seen as for Germany of the closed case CFR curves.

References

1. Ghani AC, Donnelly CA, Cox DR, Griffin JT, Fraser C, Lam TH, et al. Methods for estimating the case fatality ratio for a novel, emerging infectious disease. Am J Epidemiol. 2005;162(5):479–86.

2. Bi Q, Wu Y, Mei S, Ye C, Zou X, Zhang Z, et al. Epidemiology and Transmission of COVID-19 in Shenzhen China: Analysis of 391 cases and 1,286 of their close contacts. medRxiv. 2020;1–22.

3. Verity R, Okell LC, Dorigatti I, Winskill P, Whittaker C, Imai N, et al. Estimates of the severity of coronavirus disease 2019: a model-based analysis. Lancet Infect Dis. 2020;3099(20):1–9.
