# Supplementary material for: Analysis of the time course of COVID-19 cases and deaths from countries with extensive testing allows accurate early estimates of the age specific symptomatic CFR values
Source: PLoS One. 2021 Aug 18;16(8):e0253843. doi: 10.1371/journal.pone.0253843 (PMC8372929; doi:10.1371/journal.pone.0253843)
Supplement: S1 Table — This table shows the ratio of negative COVID-19 tests to 1 positive COVID-19 test, and the number of COVID-19 tests per 1,000,000 in the population for each of the 7 countries included in our analysis, as of May 10, 2020 [1–8]. (PDF) [file pone.0253843.s005.pdf]

S 1 Table. Ratio of total to positive tests and tests per 1,000,000 in the population. This table shows the ratio of negative COVID-19 tests to 1 positive COVID-19 test, and the number of COVID-19 tests per 1,000,000 in the population for each of the 7 countries included in our analysis, as of May 10, 2020 [1–8].

	Total Tests to Positive Tests Ratio	Tests per 1M
**Australia**	119.27	32,466
**Austria**	19.94	35,143
**Iceland**	30.09	158,816
**Israel**	27.63	52,598
**Germany**	16.04	32,891
**New Zealand**	127.39	39,468
**South Korea**	61.05	12,949

References

1. Worldometer. COVID-19 Coronavirus Pandemic. Dover; 2020.
2. Australian Government Department of Health. Coronavirus (COVID-19) health alert April 21, 2020. Canberra; 2020.
3. Federal Ministry for Social Affairs Health Nursing and Consumer Protection. Austria: Official COVID19 dashboard public information. Vienna; 2020.
4. Directorate of Health. COVID-19 in Iceland – Statistics. Reykjavik; 2020.
5. Ministry of Health. COVID-19 Update [Internet]. Government of Israel. Jerusalem; 2020. Available from: https://govextra.gov.il/ministry-of-health/corona/corona-virus/
6. Robert Koch Institute. COVID-19 in Germany. Berlin; 2020.
7. Ministry of Health. COVID-19 - current cases. New Zealand Government. 2020.
8. Central Disease Control Headquarters. Coronavirus Disease-19, Republic of Korea. Sejong; 2020.
