# Supplementary material for: Analysis of the time course of COVID-19 cases and deaths from countries with extensive testing allows accurate early estimates of the age specific symptomatic CFR values
Source: PLoS One. 2021 Aug 18;16(8):e0253843. doi: 10.1371/journal.pone.0253843 (PMC8372929; doi:10.1371/journal.pone.0253843)
Supplement: Attachment — Submitted filename: What are the main claims.docx [file pone.0253843.s006.docx]

PLOS ONE

Analysis of the time and age dependence of the case-fatality-ratio for COVID-19 in seven countries with a high total-to-positive test ratio suggests that the true CFR may be significantly underestimated for the United States in current models(Manuscript Number: PONE-D-20-16432)

Reviewer’s comments to authors:

This is an interesting article regarding the corrected CFR calculation of COVID-19 for specific countries, particularly using the data of the delayed

time from symptom to death and the age groups. Besides the manuscript title could be shorten more for capturing the article core and major contributions to the literature, I raised several concerns below, If the title is mainly focused on the CFR calculation with delayed time and the population ages.

Title: Calculation of the true case fatality rate(CFR) for COVID-19 applied to infected countries using the delayed onset-to-death time and age dependence.

Major concerns:

1. What are the main claims of the paper and how significant are they for the discipline?

This article attempts to propose one corrected CFR calculation to the literature. However, too many tables and Figures as well as Appendixes that make readers confused with a mess to read this article is the disadvantage. Usually, only five Tables and Figures are recommended in one article. Otherwise, the focus will be substantially deviated.

2. Are the claims properly placed in the context of the previous literature? Have the authors treated the literature fairly?

Numerous articles have addressed and criticized the naïve CFR lower than the true CFR at the end of the epidemic. What method or model that could be applied to estimate the TRUE CFR is required to study. Even several infected countries have experienced the COVID-19 and gained the TRUE CFR, how to model the calculation of the true CFR for infected countries using the retrospective study is merit a good investigation. However, this study cannot focus the main research study and follow an appropriate approach to report the study results for this study.

3. Do the data and analyses fully support the claims? If not, what other evidence is required?

First, one study should provide adequate data that can let the followers to mimic the study in the future. We have not seen the data regarding the age distribution in each studied countries. Furthermore, the data in Table 3, all those proportions of p(0-69). p(70-79), p(70+), and p(80+) should equal 1.0 but not in this table. For instance, the total is greater than 1.0 according to data in Australia(86.11% 10.54% 13.89% 3.35%) shown in Table 3.

Second, It is not sufficient just stating data obtained from the sites (Worldometer, Statista) or data was also obtained from the New York City Department of Health website.(17,31) in Sources of data

Third, The previous data regarding naïve CFR, age groups in population, and the time delayed in each country should be provided in this study.

4. *PLOS ONE* encourages authors to publish detailed protocols and algorithms as supporting information online. Do any particular methods used in the manuscript warrant such treatment? If a protocol is already provided, for example for a randomized controlled trial, are there any important deviations from it? If so, have the authors explained adequately why the deviations occurred?

The authors proposed detailed protocols and algorithms in the current study. However, if Bayes' theorem was applied in this study, the true CFR could be obtained for each infected country. As such, authors should express any difference from the Bayes’ theorem applied to compute the true CFR in Discussions.

5. If the paper is considered unsuitable for publication in its present form, does the study itself show sufficient potential that the authors should be encouraged to resubmit a revised version?

The study structure should be well reorganized further. For example, following the structure of Introduction, Methods, Results, Discussions and conclusion is required.

Moreover, the word of present is suggested using presence in the sentence of “by calculating the percent of the”. The paragraph of “In contrast, using previously reported IFR values gave minimum estimates between 1.5 and 10-fold higher (see Figure 7).(5,8–11,15,16)” is different from Table 1 with IFR values less than 1% when compared to those “between 1.5 and 10-fold” in the paragraph.

Why Table 1, Figure 6, Table 2 and Figure 7 are abruptly seen in Introduction instead of the number order one by one in results is concerned by readers.

Only Five Tables and/or Figures are suggested in this study for readers easy to capture the whole picture or concept of this study instead of so many in Figures or appendixes confusing readers. .

.

6. Are original data deposited in appropriate repositories and accession/version numbers provided for genes, proteins, mutants, diseases, etc.?

No, we concern the data regarding the age groups in population in each country. If Table 3 provided those to readers, but the summation of proportion in age groups is greater than 1.0. As mentioned above, the the naïve CFR and age proportion for each country have been provided, the true CFR is easy to compute when Bayes’ theorem is applied.

7. Are details of the methodology sufficient to allow the experiments to be reproduced?

No. the methodology should be easy to follow by readers. At least, I have not the feeling of easy to reproduce the study in the future.

8. Is the manuscript well organized and written clearly enough to be accessible to non-specialists?

No, the clearer and easier to read is better for readers with interest to read and cite in detail or in the future.

Summary, I suggest readers shortening the title and let readers quickly catch the whole concept and purpose of this study at a quick glance.

The number of Tables and Figures should be condensed to report the main findings in this study. Figure 6 is nice but the computation of ture CFR should be easy to follow for non-specialist
