# Supplementary material for: Analysis of the time course of COVID-19 cases and deaths from countries with extensive testing allows accurate early estimates of the age specific symptomatic CFR values
Source: PLoS One. 2021 Aug 18;16(8):e0253843. doi: 10.1371/journal.pone.0253843 (PMC8372929; doi:10.1371/journal.pone.0253843)
Supplement: Attachment — Submitted filename: Replytoreviewercoment11.1.2020.docx [file pone.0253843.s007.docx]

We thank the reviewers for their encouragement regarding the significance of the paper and for their many helpful comments and suggestions. We have extensively revised the text of the manuscript to focus on the use of the method for determining accurate values of the symptomatic CFR from data early in the pandemic. We have also simplified and clarified the descriptions of our methods and results throughout. The paper is also considerably shortened with several figures and text focusing on minor technical details removed. We have also updated our terminology for clarity and expanded our list of definitions. Revised sections of the manuscript are in blue print. Specific replies are given below in green.

**Comments to the Author**

1. Is the manuscript technically sound, and do the data support the conclusions?

The manuscript must describe a technically sound piece of scientific research with data that supports the conclusions. Experiments must have been conducted rigorously, with appropriate controls, replication, and sample sizes. The conclusions must be drawn appropriately based on the data presented.

Reviewer #1: Yes

Reviewer #2: Partly

We have clarified how our conclusions follow from our results throughout. .

2. Has the statistical analysis been performed appropriately and rigorously?

Reviewer #1: No

Reviewer #2: No

We revised our explanation of our statistical and other procedures in the Calculations and throughout.

3. Have the authors made all data underlying the findings in their manuscript fully available?

The [PLOS Data policy](http://www.plosone.org/static/policies.action#sharing) requires authors to make all data underlying the findings described in their manuscript fully available without restriction, with rare exception (please refer to the Data Availability Statement in the manuscript PDF file). The data should be provided as part of the manuscript or its supporting information, or deposited to a public repository. For example, in addition to summary statistics, the data points behind means, medians and variance measures should be available. If there are restrictions on publicly sharing data—e.g. participant privacy or use of data from a third party—those must be specified.

Reviewer #1: No

Data will be made available in CSV format once the article is accepted for publication.

Reviewer #2: Yes

4. Is the manuscript presented in an intelligible fashion and written in standard English?

PLOS ONE does not copyedit accepted manuscripts, so the language in submitted articles must be clear, correct, and unambiguous. Any typographical or grammatical errors should be corrected at revision, so please note any specific errors here.

Reviewer #1: No

We have clarified the procedures, data used, and conclusions based on the comments of the reviewers.

Reviewer #2: Yes

5. Review Comments to the Author

Please use the space provided to explain your answers to the questions above. You may also include additional comments for the author, including concerns about dual publication, research ethics, or publication ethics. (Please upload your review as an attachment if it exceeds 20,000 characters)

Reviewer #1: I suggest readers shortening the title and let readers quickly catch the whole concept and purpose of this study at a quick glance.
The number of Tables and Figures should be condensed to report the main findings in this study. Figure 6 is nice but the computation of ture CFR should be easy to follow for non-specialist

Following the guidance of the reviewer we have reduced the number of figures, modified the title, abstract and text, and condensed the reported main findings for clarity and to emphasize the importance of the CFR determination .

Reviewer #2: Summary: The authors study age and time dependence of the case fatality ratio in a handful of example countries. The questions asked by the authors are certainly important. The data is presented in a useful way and many of the conclusions reached are consistent with the best available evidence for different severity estimates of COVID. However, I believe the paper has a number of methodological and presentational shortcomings. I believe the paper is not of publishable standard currently and to be frank it would need almost a complete overhaul before it would be publishable.

Thank you for your support of the significance of the wall and support for our conclusions.

We have extensively clarified our methodological procedures and in doing so believe we have addressed the reviewers specific concerns.

Major comments:

Most of the mathematics in the text I believe needs to be re-written and re-formatted for readability, as it is currently, its incredibly hard to read as it doesn't flow. It includes many uses of plain english ("CFR", age ranges, "corrected" etc), which make it almost impossible to read. On top of that, the logic that the authors are trying to move through as they describe the methods often feels clunky. Its not completely clear why certain choices are made for example.
We have reformatted the Calculations for readability. We have also added subscripts to all of our usage of the term CFR to make clear throughout specifically what is referred to.

The methods include some overstated claims I believe. For example, the subsection called "Optimization of the parameters of f_D" I do not believe is describing parameter optimisation. I could be wrong, but based on how it is currently written, it seems as though a couple of arbitrary choices were made for medians and log SDs of the distribution and inspecting the goodness of fit. So perhaps the optimal parameters out of the three or so choices of median were determined, but the section is misleading. Why were these choices made. To be frank, I believe this style of modelling is of quite poor standard

We have changed the subsection title to: Assessment of the sensitivity of the correction factor to the assumed input function. As we clarify the choices of medians and SD values were taken from the studies available that measured them in patients in January and February. Because there was a range of values reported we examined the impact on the time to death CFR values we calculated (referred to now as CFRcrudetimecorrected.

I am very surprised that there is no mention of under-ascertainment of cases as a source of bias when estimating the CFR of COVID. It is well-known that many, even most in some countries, of symptomatic cases are (or at least have been, during peaks of certain countries epidemics) under-ascertained. This can bias estimates of the CFR, along with many other important epidemiological quantities upwards significantly. The authors must mention this at the very least. If the purpose of the paper is to provide accurate CFR estimates, the authors need to adjust for this. I don't see any attempt to do this in the current form of the manuscript.

The manuscript deals with the missed case problem in two ways, which we make clearer in the text.

First we deal with the problem of whether it is possible to apply a correction for the time delay to death early in the pandemic when the crude CFR can be artificially low. . At the time we submitted the paper this was a major question and led to confusion regarding the whether the disease was less deadly in countries such as Germany which initially had a very low crude CFR. This same confusion exists today with the resurgence of COVID cases through the United States and Europe.

We then show for the 6 countries the following

1. Using either a standard time to death correction or closed case CFR convergence it was possible early in the pandemic to accurately predict the true CFRcrude (CFRcrudecorrected)
2. Using a time to death distribution function derived earlier from clinical data in China was sufficiently robust to no have a large influence on the correction, as shown in the SI by our sensitivity analysis

Net we addressed the question of whether the large variation between countries in CFRcrudetimecorrected was due to differences in ascertainment or instead was due to the age dependence of CFRtrue. Based on our analysis the large majority of the variation could be explained by age which strongly supports that the individual countries were successfully ascertaining the large majority of their symptomatic cases.

We also add a comparison of our derived age specific CFR coefficients (CFRactual in the revised manuscript) with a recent study by Shaman and coworkers published in Lancet in which they calculated from much more extensive data the IFR (and symptomatic CFR) for NYC and a paper by Seoanes who calculated IFR values for many countries in Europe, as well as Asia, based on large published National random serology studies as well as ascertainment of deaths that were not originally reported.

The methods are often very crude. For example, dividing by 2 to move from CFR to IFR, because roughly half of the infections were asymptomatic on the Diamond Princess. The proportion of symotomatic/asymptomatic on the Diamond Princess is known and publicly available, so using the exact ratio is very easy. Furthermore, accurate inferred estimates of this proportion and its dependency on age have been estimated, using very detailed transmission models and fitted using MCMC. I can't quite understand why accurate estimates have not been used and very crude unjustified methods have been used.

Since the focus of our paper was whether it was possible to calculate accurate symptomatic CFR and secondarily IFR coefficients from early data in the pandemic we used the previous correction factor as it was the best available at the time.

We note that the correction factor was derived from several references (see text). We now report the full range reported by these references ( approximately 30 to 50% asymptomatic) and note that it is similar to the best values reported by the CDC for adults who constituted the very large majority of fatalities in the countries we obtained the coefficients from, as well as in NYC and China where we applied the coefficients to predict	their IFR.

There is somewhat of a mismatch between the detail in descriptive data and some of the estimates and the crudeness of some elements of the methods. The authors are clearly aware that accuracy is required when reporting important estimates. However, they seem far less aware that the same level of accuracy and rigor is required in the methods, especially when more accurate/rigorous approaches are not much more effort or difficult (given the inordinate of high quality studies on COVID already available).

We feel a major advantage of our method, while less sophisticated than methods that try to ascertain non ascertained cases, is it requiring just data regarding the age distribution of cases and deaths provided there is enough testing to have a high ratio of tests to positive cases. The complexity of these other methods can be a disadvantage when sufficient other data is not available, a threshold that is very hard to determine. Overall however it is clear from both our Table 1 and a more extensive meta analysis of studies at the time we performed our analysis (april through early May) that the values of the large majority of these studies often were large underestimates of what is currently the best accepted values. In contrast, as further emphasized in our new table 4, the values we determined remain very similar to best current values based on serology testing as well as more advanced case ascertainment procedures than could be applied early in the pandemic.

tc.

PLOS ONE

Analysis of the time and age dependence of the case-fatality-ratio for COVID-19 in seven countries with a high total-to-positive test ratio suggests that the true CFR may be significantly underestimated for the United States in current models(Manuscript Number: PONE-D-20-16432)

Reviewer’s comments to authors:

This is an interesting article regarding the corrected CFR calculation of COVID-19 for specific countries, particularly using the data of the delayed

time from symptom to death and the age groups. Besides the manuscript title could be shorten more for capturing the article core and major contributions to the literature, I raised several concerns below, If the title is mainly focused on the CFR calculation with delayed time and the population ages.

Title: Calculation of the true case fatality rate(CFR) for COVID-19 applied to infected countries using the delayed onset-to-death time and age dependence.

We have revised and shortened the title as suggested by the reviewer (with a minor modification) and emphasized this point throughout including in our revised Abstract, Introduction and Discussion. We have also eliminated redundant figures as well as text that focused on minor technical points to further sharpen the focus.

Major concerns:

1. What are the main claims of the paper and how significant are they for the discipline?

This article attempts to propose one corrected CFR calculation to the literature. However, too many tables and Figures as well as Appendixes that make readers confused with a mess to read this article is the disadvantage. Usually, only five Tables and Figures are recommended in one article. Otherwise, the focus will be

substantially deviated.

We have reduced the number of figures to 4. We kept the 3 tables because we feel the information they provide is important and is best summarized in tabular form. A short fourth table was added to compare our results with age specific symptomatic CFR/IFR based on subsequent large serology studies with improved information on fatalities.

2. Are the claims properly placed in the context of the previous literature? Have the authors treated the literature fairly?

Numerous articles have addressed and criticized the naïve CFR lower than the true CFR at the end of the epidemic. What method or model that could be applied to estimate the TRUE CFR is required to study. Even several infected countries have experienced the COVID-19 and gained the TRUE CFR, how to model the calculation of the true CFR for infected countries using the retrospective study is merit a good investigation. However, this study cannot focus the main research study and follow an appropriate approach to report the study results for this study.

The purpose of this paper is to demonstrate that even with minimal data early on in an epidemic, our methods were still able to calculate the true symptomatic CFR as evidenced by the results of the serology studies from New York City and our comparison in Table 4 with the best values now available from large serology studies and further ascertainment of COVID deaths.

The fact that our results are in very good agreement with these later studies, despite not having this information available supports our premise that our method, by focusing on countries that have extensive testing to determine true CFR/IFR coefficients, was effective.

3. Do the data and analyses fully support the claims? If not, what other evidence is required?

First, one study should provide adequate data that can let the followers to mimic the study in the future. We have not seen the data regarding the age distribution in each studied countries. Furthermore, the data in Table 3, all those proportions of p(0-69). p(70-79), p(70+), and p(80+) should equal 1.0 but not in this table. For instance, the total is greater than 1.0 according to data in Australia(86.11% 10.54% 13.89% 3.35%) shown in Table 3.

Table 3 has all of the data that the reviewer requests. We have clarified our description of the table as well as the terms used to make the information in the table easier to interpret

Second, It is not sufficient just stating data obtained from the sites (Worldometer, Statista) or data was also obtained from the New York City Department of Health website.(17,31) in Sources of data

We clarified the sources of data in Methods. Data will be made available in CSV format once the article is accepted for publication.

Third, The previous data regarding naïve CFR, age groups in population, and the time delayed in each country should be provided in this study.

Data will be made available in CSV format once the article is accepted for publication.

4. *PLOS ONE* encourages authors to publish detailed protocols and algorithms as supporting information online. Do any particular methods used in the manuscript warrant such treatment? If a protocol is already provided, for example for a randomized controlled trial, are there any important deviations from it? If so, have the authors explained adequately why the deviations occurred?

The authors proposed detailed protocols and algorithms in the current study. However, if Bayes' theorem was applied in this study, the true CFR could be obtained for each infected country. As such, authors should express any difference from the Bayes’ theorem applied to compute the true CFR in Discussions.

We have opted not to conduct this analysis in the Bayesian framework, as the methods we present were rigorous enough to estimate age specific true CFR values and predict accurate CFR/IFR values for regions with limited testing, as validated by later serology studies (table 4 and description)

5. If the paper is considered unsuitable for publication in its present form, does the study itself show sufficient potential that the authors should be encouraged to resubmit a revised version?

The study structure should be well reorganized further. For example, following the structure of Introduction, Methods, Results, Discussions and conclusion is required.

We had Introduction, Results, Discussion and Conclusion as sections previously, and we have added a Methods title above the “Sources of Data” header.

Moreover, the word of present is suggested using presence in the sentence of “by calculating the percent of the”. The paragraph of “In contrast, using previously reported IFR values gave minimum estimates between 1.5 and 10-fold higher (see Figure 7).(5,8–11,15,16)” is different from Table 1 with IFR values less than 1% when compared to those “between 1.5 and 10-fold” in the paragraph.

We have extensively revised the text to make comparisons clearer

Why Table 1, Figure 6, Table 2 and Figure 7 are abruptly seen in Introduction instead of the number order one by one in results is concerned by readers.

Only Five Tables and/or Figures are suggested in this study for readers easy to capture the whole picture or concept of this study instead of so many in Figures or appendixes confusing readers. .

We have reduced the number of figures to 4. We added one more table because it facilitated the comparison with present best IFR coefficients more effectively than having the numbers in the text

.

6. Are original data deposited in appropriate repositories and accession/version numbers provided for genes, proteins, mutants, diseases, etc.?

No, we concern the data regarding the age groups in population in each country. If Table 3 provided those to readers, but the summation of proportion in age groups is greater than 1.0. As mentioned above, the the naïve CFR and age proportion for each country have been provided, the true CFR is easy to compute when Bayes’ theorem is applied.

We have clarified the age group related coefficients in the table.

. The p() categories overlap in terms of age groups, so only certain combinations will sum to 1. We have opted not to do this analysis in the Bayesian framework.

7. Are details of the methodology sufficient to allow the experiments to be reproduced?

No. the methodology should be easy to follow by readers. At least, I have not the feeling of easy to reproduce the study in the future.

Data will be made available in CSV format once the article is accepted for publication.

8. Is the manuscript well organized and written clearly enough to be accessible to non-specialists?

No, the clearer and easier to read is better for readers with interest to read and cite in detail or in the future.

We have made edits to make our wording clearer.

Summary, I suggest readers shortening the title and let readers quickly catch the whole concept and purpose of this study at a quick glance.

The number of Tables and Figures should be condensed to report the main findings in this study. Figure 6 is nice but the computation of ture CFR should be easy to follow for non-specialist

We thank the reviewer again for their very helpful suggestions and have incorporated the majority in the revised version
