# Supplementary material for: Analysis of the time course of COVID-19 cases and deaths from countries with extensive testing allows accurate early estimates of the age specific symptomatic CFR values
Source: PLoS One. 2021 Aug 18;16(8):e0253843. doi: 10.1371/journal.pone.0253843 (PMC8372929; doi:10.1371/journal.pone.0253843)
Supplement: Attachment — Submitted filename: Reviever response 2.28.21DLRJR.docx [file pone.0253843.s008.docx]

In summary, this article is of importance in particular comparison of CFR and IFR made among countries during the COVID-19 epidemic. For improving the readability of this article, several questions should be classified further before consideration of publication in the journal of PLOS ONE.

We thank the reviewer for their careful critical reading of the paper and the support for its significance. We have made major changes to the manuscript to strengthen the paper based on the reviewer’s concerns which we summarize below.

Major concerns:
1. It is hard to read this article for reviewers when no double spaces remained between lines.

We have made the article double-spaced.

2. Why manuscript cannot be as abstract with the formal structure of Background(or Introduction in context), Methods, Results, and Discussion(or plus condition in context). The Sources of data after Introduction is weird and rare in the format of formal scientific articles.

We have moved the sources of data to the Methods section.

3. In line 3 in the Introduction, (referred to as the case fatality ratio, CFR) and infected cases (IFR) instead of infected fatality ratio (IFR) used in this study.

We make clear throughout the revised manuscript that CFR is symptomatic case fatality ratio and IFR infected fatality ratio. We also use subscripts to clarify the different uses of CFR (crude, timecorrected, actual) and provide definitions.

4. At the first time to report the terms such as IFT and CFR that should be defined clearly as those in WIKI at <https://en.wikipedia.org/wiki/Case_fatality_rate>

We added the following sentence on pg 3 lines 5-8: “The CFR is the number of deaths divided by the number of symptomatic cases in a given time period, and the IFR is the number of deaths divided by the number of infected cases (i.e. cases that may or may not be symptomatic) in a given time period.”

5. In the Introduction, we have not been aware of the reason for conducting this study and the purpose of this study. Using Table 1 in the Introduction is rare in the scientific article. How to make a concise introduction for readers to capture the motivation and purpose of this study is of importance. For instance, using the correct CFRs with each other in countries is essential and necessary.

In the revised Introduction we emphasize the reasons for conducting the study and purpose. We agree with the reviewer about how essential and necessary having accurate CFR values are.

We establish the motivation for this study as: “. Knowing the fraction of individuals infected with COVID-19 who will die or require hospitalization is critical for epidemiological modeling and public health policy for mitigating the disease.” We further expand upon the motivation for this study on pg. 3 lines 2-20.

We included Table 1 in the introduction because we believe that the large range of reported IFR and CFR values presented demonstrate the need to develop improved approaches for rapidly determining these values without having to wait until the end of the outbreak for retrospective analysis.

6. It is important for readers who are able to replicate a similar study in the future. We have not seen any that could help readers redo the study in future studies.

Although authors addressed that the dataset can be provided after acceptance in the publication in response to the reviewer’s comments, the focus is not on the total fully dataset, but the calculation or process of the model parameter estimation that readers hope to understand even a partial or piece of data is enough. In this revised manuscript, I have also not been aware of any about the correct CFR calculation that can be understandable for readers.

The dataset (all data that was used in the equations) will be available as a csv.

The revised methods section presents the complete procedures used for determining the true CFR value from reported deaths and infections per day.

Briefly we present two methods of calculating the actual CFR from data obtained early in an outbreak: 1) a time corrected crude CFR and 2) a closed case CFR.

For the time corrected CFR, we used a lognormal distribution of percent deaths per day after diagnosis obtained directly from published patient studies early in the pandemic from China. The early studies were chosen because they were the only available at the time of our analysis and furthermore they provided a better test of our hypothesis that it is possible to obtain accurate corrections even early in a pandemic.

We then evaluated the fit to the data for the countries analyzed using the range of parameters reported in the literature. We found the fits were relatively insensitive to the reported range of values. The values that gave the best fit were approximately the average of reported values with logSD of 0.5 and median days of 14 which equates LogMu(14) = 2.64 (This is given on pg. 11 lines 6-14, pg 12 lines 1-11).

We provide the steps for this time adjustment corrected CFR (CFR_crudetimecorrected_) on pg. 9 lines 12-23, pg. 10 lines 1-18.

The second method using the closed case CFR is presented on pg. 10 lines 20-23, pg. 11 lines 1-4 and is defined as: Same as CFR_crude_ but measured using only data from closed cases (either recovered or dead) given by [ND(t)/NCC(t)].

7. In Sources of data, the definitions of symbols in equations should be followed after the formula or equation. For instance, the symbol of I for Infection: individuals who are symptomatic or asymptomatic has not found any in the formula. Keep in mind, authors should make efforts to let readers replicate and understand the study completely. Otherwise, all study are in vein. Similarly, the structure of manuscript that should be in accordance with the common style and format is necessary to be similar to (or at least not different from) other’s thinking process. For example, the section on Optimization of the parameters of fD should let us know how to obtain the fD rather than using the narrative only in the paragraph, but describing what software or statistics used in this study. That is, we are interested in this part of the study because it is important in epidemiology.

An example that can be illustrated in this study is wonderful to make readers easy capture the core of this study. It is a pity to waste the efforts made by the authors in this study because the calculation or parameter estimation is not clear and not understandable. Any result or discussion in Results and Discussion to explain the reason for the gap and difference in real-world is unnecessary.

We have restructured the paper to follow the standard format of Introduction, Methods, Results, and Discussion. We also extensively rewrote the paper to make the motivation for performing it clear, as well as the steps we took in the analysis and why.

8. I appreciate this study topic that is of interest and importance. Hopefully, this major concern can be improved in the next run of manuscript submission.

We thank the reviewer for their encouragement and interest in the study and incorporated all of their very helpful suggestions.

Minor concerns:

We have extensively rewritten the paper to address all of the concerns below.

Grammar to be improved
In Abstract:
age dependent CFR time courses to explain this increase and to
- age-dependent CFT……..
Age dependent time to fatality corrected CFR was calculated using two independent
- age-dependent time to fatality……..

A linear model was developed that predicts CFR based on age dependent CFR
-age-dependent CFR……..

coefficients and the age distribution of cases. The model was tested by a linear regression of each country’s CFR against case percentage of 70 years and over.
- by linear regression
The linear model based on their age specific CFR values provides an alternative
- age-specific CFR

Introduction:
For the United States and United Kingdom, even lower true CFR and IFR values have been reported
--- the United Kingdom

reexamined the reported number of fatalities and nCFR values outbreaks on May 7, 2020 for
-2020, for
Australia, Austria, Germany, Iceland, Israel, New Zealand, and South Korea. In all cases these countries continued to have a high degree of testing and tracking and testing of contacts as
- In all cases, these……
United States and 8:1 for the United Kingdom, (Appendix 4). Therefore, their final reported
- the United Kingdom (Appendix 4)

CFR values even early in the outbreaks when the reported nCFR values were several fold lower.
-several folds lower
for the United States and United Kingdom.(5,10,13,14) Despite this range, the large
- the United Kingdom


From this model we estimated the corrected CFR for China using the linear model from the case
- From this model,

in total number of fatalities recently reported by the Chinese government.(2)
Regarding US and UK populations, there is a much wider range of CFR and IFR estimates, with
-- A total number of …….

We therefore tested our model by calculating the percent of the population who has been
-, therefore,


onset (day of positive test) to fatality distributions for Chinese patients outside of Wuhan who
A positive test

censoring (fatalities missed due to the limited patient observation time). The best fitting distributions from these sources were very similar, with Linton reporting a best fit median of
- best-fitting
13.2 days with a 95% CI of 11.5 to 15.3 days, and Mizumoto et al. reporting a best fit median
- the best fit median
all studies, based on gamma fits, was very similar, and equivalent to a logSD of approximately
--- very similar and equivalent

Goodness of fit was then determined by calculating the least squares total residual - The goodness of fit was….
the population and the age specific CFR values. Studies have reported that the nCFR - age-specific DFR
COVID-19 strongly increases with age.(5–7,9–11,15,21,22,32–34) We determined for
country the corrected CFR values for the fraction of a country’s populations of age
- for a fraction -

cumulative fatalities (Appendix 5). Case per day data from South Korea and Germany - per day of data from…..

shifted several days later and a less right skewed distribution.(1,5,32) Based on these - right-skewed distribution


population infected, we then divided the maximum and minimum number of
- and a minimum number of

due to the random testing not including children, who are known to have a much –
- , not including children,

plot using the South Korean data, which rose from a low of 0.55% on March 8, 2020 to its value
-2020,
on May 7, 2020 of 2.28%. The values shown are plotted from 10 days after the first 2020,
were reported to avoid large fluctuations, these due to the low number of fatalities - fluctuations due to the low number of

Figure 1. Reported nCFR(t) increases with time after outbreak for Germany. The nCFR the outbreak for Germany
continuously increased with time after the outbreak, from a lowest value of 0.12% on March 10, 2020 to
-2020,
its present value on May 7, 2020 of 4.36%. The dashed horizontal line at 5.0% is our -2020,

CFR from the closed case CFR value.
Figure 2. Reported nCFR(t) versus day after outbreak for South Korea. The nCFR for South
Korea is also seen to be continuously increasing from a minimum of 0.55% on March 8, 2020 to 2.28%
-2020,

curve, the closed case fatality ratio appears to be converging to a near constant value. In contrast the
a near-constant
mean of
0.129- 0.204, which is within error the same as the slope determined by linear regression value.
-the liear regression value

cCFR70+ + cCFR69. It is seen that for all countries the cCFR70 term explains the large - for all countries,

the New York City population that has been infected by COVID-19 up through April 22, 2020 in order to compare with recent studies that have performed random -0
-2020,

College model, the estimated percentages of the population infected are several fold above the
-several-fold

corrections for missed
cases.(3–11,20–22,34) We found that in all cases there was a several fold increase in -that in all cases, several-fold
n
between diagnosis and fatality. Despite the high level of testing we found a wide ---
-f testing,

When we examined the component of the CFR due to this population (cCFR70+) ---
-componence of

April 22, 2020 was 2 to 16.5-fold higher than previous values that have been applied -2020,

between 70 and 79 and 80 and over years old, but higher than the majority but not - over the years old


least several fold from the early values used to justify low estimates of the IFR for COVID-19
-several-fold
(see Figures 5A, 5B, and Appendix 3).(12,14,24–27,30) We therefore calculated the -, therefore,


We did not factor in preexisting conditions in our analysis which has been reported
- missing verb????

fraction of the case population in the 60 – 69 group which also has a highly elevated -, which also has a highly
27) However due to the low number of fatalities in several countries in the 0- 69 age -. However,
did not perform a sub analysis.
-sub-analysis.

asymptomatic. This value may be an overestimate, as shown by Mizumoto, because - by Mizumote because…..


possibility in which none of the active cases as of April 22, 2020 subsequently died ---
-2020,

COVID-19 (33 out of 215 having the virus).(16) In the second study the New York City -in the second study,

of time, and corrected for age distribution of positive cases.(11) No time correction - for the age distribution
