# Supplementary material for: Analysis of the time course of COVID-19 cases and deaths from countries with extensive testing allows accurate early estimates of the age specific symptomatic CFR values
Source: PLoS One. 2021 Aug 18;16(8):e0253843. doi: 10.1371/journal.pone.0253843 (PMC8372929; doi:10.1371/journal.pone.0253843)
Supplement: Attachment — Submitted filename: Reviewer response 5.4.21DLR.docx [file pone.0253843.s009.docx]

Reviewer #4: This study provides an important method to predict COVID-19 infection by calculating age specific symptomatic CFR values. However, the whole article is not well organized to show the significant finding:

*We thank the reviewer for their support of the importance of the findings. We have adopted all of their suggestions to improve the organization and overall clarity for the reader. In the revised text we use red font to indicate sections with substantial changes addressing the reviewer’s points. We also made minor changes in the Methods and Discussion for clarity.*

1. The introduction in current status need to revised to illustrate the importance, necessity and motivation of estimates of the age specific symptomatic CFR values. Also, using table in the introduction is not a common practice for scientific papers. Please move it to result or supplementary section.

*We have moved the description of the meta analysis and the table to the Results section.*

2. The procedures 1-6 is more like methods, please simplify this part and introduce the most important information or findings.

*We removed procedures 1 – 6 from the Introduction. We also modified the Introduction to end with a standard statement of the most significant findings.*

*However, because a point by point overview of the procedures was requested by a previous*

*reviewer we moved them to the beginning of the Methods section.*

*The following sections in Methods still provide detailed descriptions of each procedure. However, per the reviewer’s suggestion, we emphasize where standard methods were used.*

3. It’s easier for reader to detail the statistics method and software used in this study, instead of using mathematical formula. For example, what kind of regression were used to validate age dependent CFR_crudetimecorrecte_d values.

*We have added that all analyses were conducted in R and all plots were done in ggplot2 (pg. 7 lines 5-6) which is a standard software used in the field.*

*We added “linear regression” to provide more clarity as to the type of regression (pg.12 lines 4-9). We further clarified that we used simple linear regression to validate age dependent CFR_crudetimecorrected_ values (Pg. 12 lines 11-22).*

*We however have kept the equations we used in the Methods section based upon the request of earlier reviewers, who felt that the specificity was important due to variations in the methodology within the field.*

4. The figure and table should use canonical standards for scientific articles. For example, There is no necessary to mark each day in the horizontal axis in figure 1 and 2.

*We have updated Figures 1 and 2 so that every 5 days is marked instead of everyday. Also, we performed the plots in R using ggplot2 (pg. 7, lines 5 – 6) which is a standard plotting program.*
